# Mapping multi-regional functional connectivity of astrocyte-neuronal networks during behaviors

**DOI:** 10.1117/1.NPh.11.4.045010

**Published:** 2024-11-15

**Authors:** Haoyu Wang, Mingzhu Huang, Shaofan Yang, Jiameng Xu, Jin Li, Han Qin, Shanshan Liang, Teng Teng, Chuanyan Yang, Mingyue Gong, Yong He, Xingyi Li, Huiquan Wang, Xiang Liao, Xiaowei Chen, Zhiqi Yang, Kuan Zhang

**Affiliations:** a Third Military Medical University, Brain Research Center and State Key Laboratory of Trauma and Chemical Poisoning, Chongqing, China; b Chongqing University, College of Bioengineering, Chongqing, China; c Tiangong University, The School of Control Science and Engineering, Tianjin, China; d Chongqing Institute for Brain and Intelligence, Guangyang Bay Laboratory, Chongqing, China; e Peking university, School of Electronics, Beijing, China; f Chongqing University, School of Medicine, Center for Neurointelligence, Chongqing, China; g Gansu Provincial Central Hospital, Department of Neurology, Lanzhou, China

**Keywords:** multi-fiber photometry, Ca^2+^ transients, astrocyte-neuronal networks, multiple brain regions, freely moving

## Abstract

**Significance:**

Diverse behaviors rely on coordinated activity and multi-regional functional connectivity within astrocyte-neuronal networks. However, current techniques for simultaneously measuring astrocytic and neuronal activities across multiple brain regions during behaviors remain limited.

**Aim:**

We propose a multi-fiber solution that can simultaneously record activities of astrocyte-neuronal networks across multiple regions during behaviors.

**Approach:**

We employed cell-specific dual-color genetically encoded calcium indicators (GECIs) and multi-fiber photometry to simultaneously measure astrocytic and neuronal Ca^2+^ transients across multiple brain regions in freely behaving animals.

**Results:**

Our findings demonstrate that both movements and sensory stimuli induce synchronized and highly correlated Ca^2+^ transients in astrocytes and neurons of freely behaving mice. In addition, we recorded astrocytic and neuronal Ca^2+^ transients from multiple brain regions during mouse behaviors. Our observations reveal heightened synchronization of astrocytic and neuronal Ca^2+^ transients across different brain regions during movements or sensory stimuli, indicating enhanced functional connectivity within brain-wide astrocyte-neuronal networks.

**Conclusions:**

Multi-fiber photometry, combined with cell-specific dual-color GECIs, represents a powerful approach for investigating astrocytic and neuronal activities across different brain regions during behaviors. This technique serves as a versatile tool for analyzing the multi-regional functional connectivity map of astrocyte-neuronal networks associated with specific behaviors.

## 1 Introduction

At the population level, astrocytes dynamically interact with neurons.^1^^–^^3^ Astrocytes sense activity within the neuronal network and respond to neural signals by generating ${\mathrm{Ca}}^{2+}$ activities *in vivo*.^4^^,^^5^ Simultaneously, astrocyte activation or manipulation modulates neuronal network activity.^2^^,^^3^ This bidirectional, interconnected communication between astrocytes and neurons is collectively termed the “astrocyte-neuronal network.”^6^^,^^7^ Accumulating experimental evidence suggests that various behaviors result from coordinated activity between neurons and astrocytes within these networks.^2^^,^^8^^,^^9^ Therefore, deciphering the interaction between astrocytes and neurons within astrocyte-neuronal networks is essential for comprehensively understanding the genesis and dysregulation of behavior.^2^^,^^3^^,^^10^

Neurons and astrocytes in the brain exhibit diverse structures, functions, and molecular compositions depending on their respective regions.^11^^–^^14^ These diversities enable them to modulate and support neural circuits in a region-specific manner.^15^ The brain comprises multiple distinct regions, each characterized by unique combinations of neuronal subtypes and astrocytic populations.^12^ Previous studies have revealed that astrocyte-neuronal networks can be regionally regulated by noradrenergic terminals.^16^ However, the mechanisms by which astrocyte-neuronal networks employ region-specific strategies to regulate particular circuits remain not fully understood. Furthermore, behaviors critically depend on the synchronized activity of distinct brain regions.^17^^,^^18^ Recent studies indicate that neurons across various brain areas interact simultaneously and function in parallel during a wide range of behaviors.^17^ Astrocyte populations in different brain regions can coordinately respond to activity from long-range neuronal projections, thereby influencing brain states and complex behaviors.^3^ Nevertheless, it remains uncertain whether astrocyte-neuronal networks can generate correlated activity patterns across different regions and facilitate effective communication between them. Therefore, real-time measurement of astrocyte-neuronal network signals from multiple brain regions is essential for understanding the brain as a dynamic system and fully comprehending how circuit activity patterns relate to behavior.

Fiber photometry uses a fiber optic cannula to monitor fluorescent signals.^19^ When combined with genetically encoded calcium indicators (GECIs), it enables real-time monitoring of the population activity of specific cell types in freely behaving mice.^20^^–^^22^ Researchers have employed fiber photometry to explore diverse forms of activity in the brain, including the activity of specific types of neurons, as well as astrocytic activity and neurotransmitter/neuropeptide signals.^19^ Multi-color fiber photometry is particularly useful for simultaneously monitoring the activities of distinct types of cells.^21^^,^^23^^,^^24^ Using green and red GECIs, this technique can measure activities from either two types of neurons^23^^,^^24^ or from neurons and astrocytes simultaneously.^21^ Moreover, multi-fiber photometry allows the recording of fluorescent activity signals from multiple brain regions in the same freely behaving animal.^21^^,^^23^^,^^25^ This technique provides a comprehensive view of neural network activity and helps investigate functional connectivity between brain regions.^25^ Researchers have utilized dual-site fiber photometry to simultaneously monitor astrocytic ${\mathrm{Ca}}^{2+}$ activities across two brain regions.^16^^,^^26^ They have observed highly correlated astrocytic ${\mathrm{Ca}}^{2+}$ activities in response to locomotion in these regions,^26^ as well as regionally regulated astrocyte-neuronal networks.^16^ However, current methods for simultaneously measuring astrocytic and neuronal activities across multiple brain regions during behavior are limited. Developing a method to address these limitations will be crucial for fully elucidating the role of the astrocyte-neuronal network in the global brain circuit during complex behavioral processes.

In this study, we present a novel approach that combines cell-specific dual-color GECIs with multi-fiber photometry to simultaneously monitor astrocytic and neuronal ${\mathrm{Ca}}^{2+}$ activities across multiple brain regions in freely behaving mice. Our results validate the effectiveness of this method in investigating the complex interactions between astrocytes and neurons within and between different brain areas during natural behaviors. This technique enables researchers to map and analyze the functional connectivity between astrocytes and neurons across various brain regions while animals perform specific behavioral tasks.

## 2 Methods

### 2.1 Animals

C57BL/6 mice were sourced from the Laboratory Animal Center at the Third Military Medical University. We utilized 3- to 5-month-old C57BL/6 mice for our study. The mice were group-housed under a 12-h light/dark cycle and had ad libitum access to food and water. Mice implanted with optical fibers were individually housed. All animal experiments adhered to the guidelines set forth by the Institutional Animal Care and Use Committee of the Third Military Medical University, China (Animal Ethical Statement NO. AMUWEC20230126).

### 2.2 Viral Vector

The viral vectors used in this study included AAV5-GfaABC1D-cyto-GCaMP6f (52925, titer $\geq 7\times 10^{12}\;\mathrm{vg}/\mathrm{mL}$, Addgene, United States), AAV5-GfaABC1D-Lck-GFP-SV40 (105598, titer $\geq 7\times 10^{12}\;\mathrm{vg}/\mathrm{mL}$, Addgene, United States), AAV9-hSyn-NES-jRGECO1a-WPRE-SV40 (100854, titer $\geq 1\times 10^{13}\;\mathrm{vg}/\mathrm{mL}$, Addgene, United States), and AAV9-hSyn-NES-mCherry-WPRE-SV40 (114472, titer $\geq 1\times 10^{13}\;\mathrm{vg}/\mathrm{mL}$, Addgene, United States).

### 2.3 Stereotactic Virus Injection

The mice underwent anesthesia with 1% to 2% isoflurane in oxygen and were subsequently positioned in a stereotactic head frame on a heating pad (37.5°C to 38°C), following previously established protocols.^20^ After exposing the skin, the skull was uncovered, and a small craniotomy (0.5×0.5  mm) was performed directly over the injection site. We utilized specific coordinates for injections: S1 [anterior-posterior (AP) −0.5  mm, mediolateral (ML) −1.9  mm, dorsoventral (DV) −0.65  mm], M1 (AP 0 mm, ML +1.0  mm, DV −0.65  mm), and CA1 (AP −2.1  mm, ML +1.55  mm, DV −1.19  mm). All injections were administered using a glass micropipette with a tip diameter of 10 to 20  μm, securely attached to a 5-μl syringe (#75, Hamilton, United States). The injection volume (150 nl) and flow rate ($1\;\mathrm{nl}\;s^{-1}$) were controlled via a syringe pump (788130, KD Scientific, United States). After each injection, the micropipette remained in place for 10 min before withdrawal. The scalp incision was closed with tissue glue (1469SB Vetbond, 3M Animal Care Products, United States), and post-injection analgesics were provided for 3 days to aid in recovery. A complete list of all vectors used is detailed above.

### 2.4 Fiber Photometry System

We utilized the R820 dual-color multi-fiber photometry system (RWD Life Science, China) to simultaneously record astrocytic and neuronal ${\mathrm{Ca}}^{2+}$ transients. This system includes a fluorescence cube, spectrometer, and excitation light components. GCaMP6f fluorescence was excited with a 470-nm light-emitting diode (LED), while jRGECO1a fluorescence was excited with a 560-nm LED.^27^ A sequence of dichroic mirrors within the fluorescence cube directed the emitted dual-color light, which was focused through a 20× objective at the optic fiber’s end.^20^^,^^23^ The dual-color fluorescence emission was then directed back through the fluorescence cube and focused onto a lens.^20^^,^^23^ A spectrometer with a dichroic mirror separated the emission into green and red light, which were detected simultaneously by two complementary metal oxide semiconductor (CMOS) sensors. The collected dual-color fluorescence signals were digitized at a stable frequency.^20^^,^^23^

### 2.5 Fiber Photometry Recordings in Freely Behaving Mice

Mice injected with viral vectors in the S1, M1, and CA1 regions were anesthetized with 1% to 2% isoflurane and positioned in a stereotactic head frame (68001, RWD Life Science, China) atop a heating pad (37.5°C to 38°C).^20^ Ferrules (907-03007-00, outer diameter: 1.25 mm, core: 200  μm, NA: 0.39, RWD Life Science, China) were implanted 0.1 mm above the virus injection sites and secured to the mouse skull using dental cement. To simultaneously record ${\mathrm{Ca}}^{2+}$ transients in astrocytes and neurons across S1, M1, and CA1, bundled optical fibers with ceramic sleeves (1×3, fiber core: 200  μm diameter, NA 0.37, connectors: 1.25 mm, Inper, China) were connected to the ferrules fixed on the head. Mice were placed in a white square recording chamber (40×40  cm) to allow free movement during data collection. ${\mathrm{Ca}}^{2+}$ signals and behavioral videos were captured simultaneously for 45 min. Each channel of ${\mathrm{Ca}}^{2+}$ signals was digitized at 30 Hz using fiber photometry recording software (OFRS, RWD Life Science, China). Behavioral videos synchronized with ${\mathrm{Ca}}^{2+}$ signals were recorded by a camera (MV-SUA502C-T, Mind Vision, China) at 30 Hz, with a spatial resolution of 1280×720  pixels. Offline, all ${\mathrm{Ca}}^{2+}$ signals and behavioral videos were synchronized using event markers. For footshock experiments, mice were placed in a rectangular chamber where they could freely move. Footshocks (0.6 mA, 1 s) were administered every 5 min. Each mouse underwent continuous recording for four trials to observe footshock-induced astrocytic and neuronal ${\mathrm{Ca}}^{2+}$ transients.

### 2.6 Histology and Confocal Imaging

Following the experiments, we anesthetized the mice with phenobarbital sodium (1  g/kg) and performed transcardial perfusion with 4% paraformaldehyde (PFA) in phosphate buffered saline (PBS). The brains were subsequently dehydrated in 15% sucrose PFA solution for 24 h and sectioned into 40  μm coronal slices. These slices were stained with 4′,6-diamidino-2-phenylindole (DAPI) to visualize the nuclei. Imaging was conducted using a confocal microscope (LSM 980, Zeiss, Germany) equipped with a × 20 air objective (NA 0.85) and a × 40 oil immersion objective (NA 1.25), scanning at a resolution of 1024×1024  pixels with 4  μm increments in Z-stack. For immunohistochemistry, we employed the following primary antibodies: rabbit anti-NeuN (177487, 1:500, Abcam, United Kingdom), rabbit anti-S100β (287003, 1:500, SYSY, Germany), and chicken anti-GFP (ab13970, 1:500, Abcam, United Kingdom). Secondary antibodies used were Alexa Fluor 488 donkey anti-chicken (A78948, 1:800, Invitrogen, United States) and Alexa Fluor 647 donkey anti-rabbit (A-31573, 1:500, Invitrogen, United States).

### 2.7 Data Analysis

We sampled astrocytic ${\mathrm{Ca}}^{2+}$ transients at a wavelength of 470 nm and neuronal ${\mathrm{Ca}}^{2+}$ transients at a wavelength of 560 nm using optic fiber-based recordings in behaving mice. Z-scored ${\mathrm{Ca}}^{2+}$ values for both astrocytes and neurons were computed using the formula: $Z\;\text{score}=(\Delta F/F-(\Delta F/F{)}_{\text{mean}})/(\Delta F/F{)}_{\mathrm{std}},\Delta F/F=(F_{\text{raw}}-F_{\text{baseline}})/F_{\text{baseline}}$.^27^^,^^28^ Regions of interest were selected on the grayscale image of the optic fiber bundle, and the average pixel intensity was calculated as a measurement of the raw ${\mathrm{Ca}}^{2+}$ signal, denoted as $F_{\text{raw}}$. Subsequently, the OFRS software (RWD Life Science, China) was utilized with a moving window to obtain the average of $F_{\text{raw}}$, referred to as $F_{\text{baseline}}$. To analyze the data, we converted image frames to binary format to delineate mouse shapes based on image intensities and quantified mouse movement relative to body size from video clips. Traces of astrocytic and neuronal ${\mathrm{Ca}}^{2+}$ transients were examined concerning the onset of movement or footshock. Data analysis was conducted using custom-written software in MATLAB 2018b.^29^

### 2.8 Statistical Analysis

We expressed the data as the mean ± standard error of mean (SEM). Investigators conducted data analysis while blinded to group allocation. Two test methods were employed: the Wilcoxon’s signed-rank test for paired two-group comparisons and the Wilcoxon rank-sum test for unpaired two-group comparisons.^20^^,^^29^ To assess the correlation of astrocytic or neuronal ${\mathrm{Ca}}^{2+}$ transients among the S1, M1, and CA1 regions, we employed the Spearman correlation test. In addition, to examine the correlation between astrocytic and neuronal ${\mathrm{Ca}}^{2+}$ transients, we utilized the cross-correlation test.

## 3 Results

### 3.1 Dual-Color Genetically Encoded Calcium Indicators Exhibit Specific and Efficient Expressions in Both Astrocytes and Neurons

GECIs can monitor specific cell types in the brain.^30^ Using dual-color GECIs that emit at different wavelengths, we can simultaneously track ${\mathrm{Ca}}^{2+}$ transients in astrocytes and neurons.^21^ Recently, researchers used the green-shifted GECI GCaMP6f alongside red probes such as jRGECO1a to detect ${\mathrm{Ca}}^{2+}$ transients in different cell populations simultaneously.^21^^,^^23^^,^^24^ In our study, we used GCaMP6f and jRGECO1a, differentially expressed in astrocytes and neurons, to effectively achieve dual-color monitoring of both astrocytic and neuronal ${\mathrm{Ca}}^{2+}$ transients concurrently.

**Fig. 1:**
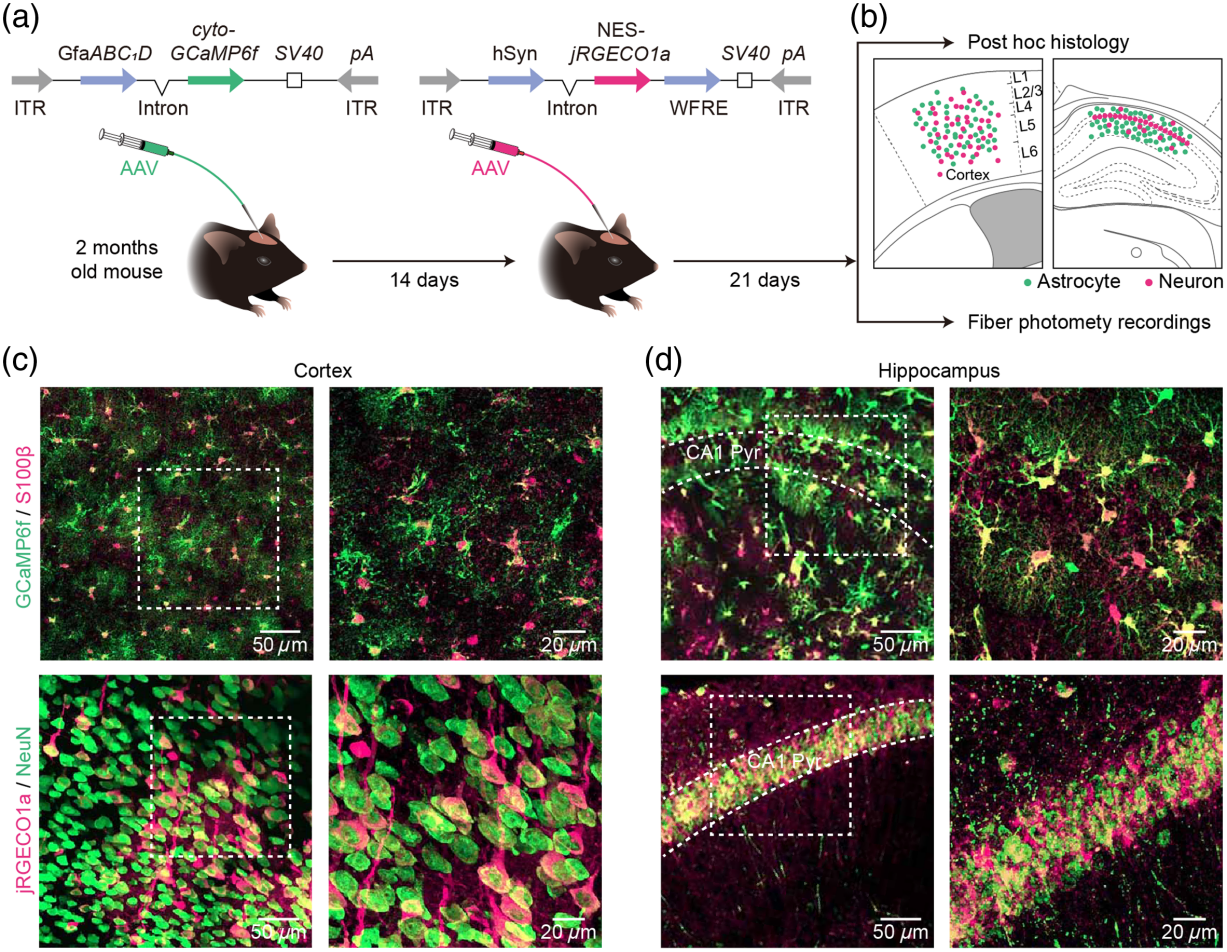
Specific and efficient expression of GCaMP6f in astrocytes and jRGECO1a in neurons within the cortex and hippocampus. (a) Top, design of AAV5 constructs to deliver cyto-GCaMP6f and AAV9 constructs to deliver NES-jRGECO1a; Bottom, protocol for AAV microinjections and the sequence of experimental approaches. (b) Top, *post hoc* analyses to identify the expression of GCaMP6f in astrocytes (green) and jRGECO1a in neurons (magenta) within the cortex (left) and hippocampal CA1 region (right). Bottom, fiber photometry recordings performed to monitor astrocytic and neuronal ${\mathrm{Ca}}^{2+}$ transients following viral injection. (c) Top, representative confocal images showing GCaMP6f (green) and S100β (magenta) immunostaining in mouse cortex after virus injections. Bottom, representative confocal image showing jRGECO1a (magenta) and NeuN (green) immunostaining in mouse cortex after virus injections (left, low power; right, high power). (d) Top, representative confocal images showing GCaMP6f (green) and S100β (magenta) immunostaining in mouse hippocampal CA1 region after virus injections. Bottom, representative confocal images showing jRGECO1a (magenta) and NeuN (green) immunostaining in mouse hippocampal CA1 region after virus injections (left, low power; right, high power).

In our research, we labeled astrocytes with GCaMP6f and neurons with jRGECO1a. Initially, we administered AAV5-GfaABC1D-cyto-GCaMP6f-SV40 to the specific brain region. Fourteen days later, we injected AAV9-hSyn-NES-jRGECO1a-SV40 into the same brain region [Fig. 1(a) and Fig. S2(a) in the Supplementary Material, strategy 1]. Twenty-one days after the AAV microinjection, we confirmed the expressions of GCaMP6f and jRGECO1a by *post hoc* histology. We then conducted fiber photometry recordings based on the locations of GECI expression in the subsequent experiments [Fig. 1(b)]. Our findings demonstrated that the above strategy for virus injections [Fig. 1(a)] led to consistent, robust, and specific expressions of GCaMP6f and jRGECO1a in the cortices and hippocampal CA1 region (CA1) of adult mice [Figs. 1(c) and 1(d), Fig. S2(b) in the Supplementary Material]. The GCaMP6f expression closely overlapped with the astrocytic marker S100β, and the jRGECO1a expression closely overlapped with the neuronal marker NeuN [Figs. 1(c) and 1(d)]. Statistical analysis revealed that GCaMP6f expression specificity was 98.2±0.7% in the cortex and 92.3±1.6% in the CA1 region (Table 1). Furthermore, the expression efficiency of GCaMP6f was 90.0±0.7% in the cortex and 98.8±0.9% in the CA1 region (Table 1). By contrast, jRGECO1a expression specificity was 77.5±1.8% in the cortex and 99.0±0.5% in the CA1 region (Table 1), with an expression efficiency of 61.9±2.0% in the cortex and 95.4±0.7% in the CA1 region (Table 1). Further experiments demonstrated that no $\mathrm{jRGECO}1a^{+}$ cells tested positive for S100β, indicating that these ${\mathrm{jRGECO}1a}^{+}$ ${\mathrm{NeuN}}^{-}$ cells are not astrocytes [Figs. S1(a) and S1(b) in the Supplementary Material]. This finding suggests that NeuN may not label all neurons. The results confirmed the specific and efficient expressions of the dual-color GECIs in astrocytes and neurons within both cortices and CA1 regions.

**Table 1:** Specificity and efficiency of GCaMP6f and jRGECO1a labeling.

Specificity (%/section)	Cortex	CA1
%S100β^+^GCaMP6f^+^/GCaMP6f^+^	98.2 ± 0.7 (n = five mice)	92.3 ± 1.6 (n = five mice)
%NeuN^+^jRGECO1a^+^/jRGECO1a^+^	77.5 ± 1.8 (n = five mice)	99.0 ± 0.5 (n = five mice)
Efficiency (%/section)	Cortex	CA1
%S100β^+^GCaMP6f^+^/S100β^+^	90.0 ± 0.7 (n = five mice)	98.8 ± 0.9 (n = five mice)
%NeuN^+^jRGECO1a^+^/NeuN^+^	61.9 ± 2.0 (n = five mice)	95.4 ± 0.7 (n = five mice)

Considering the evident leakage observed in astrocytes during transgenic labeling in previous studies,^31^ we explored two alternative virus injection strategies [Figs. S2(c) and S2(e) in the Supplementary Material, strategies 2 and 3]. One approach involved injecting a mixture of viruses containing GCaMP6f and jRGECO1a [Fig. S2(c) in the Supplementary Material, strategy 2], while the other approach entailed injecting the jRGECO1a virus first, followed by the GCaMP6f virus 14 days later [Fig. S2(e) in the Supplementary Material, strategy 3]. The results indicated that co-staining between GCaMP6f and jRGECO1a was observed in both strategies 2 and 3 [Figs. S2(d) and S2(f) in the Supplementary Material]. The leakage rate was measured at 3.1±0.8% for strategy 2 and 3.1±0.2% for strategy 3. Notably, in strategy 1 [Fig. S2(a) in the Supplementary Material], there was no co-staining between GCaMP6f and jRGECO1a, indicating the absence of leakage [Fig. S2(b) in the Supplementary Material]. In conclusion, when compared with strategies 2 [Fig. S2(c) in the Supplementary Material] and 3 [Fig. S2(e) in the Supplementary Material], strategy 1 [Fig. S2(a) in the Supplementary Material] emerged as the optimal virus injection approach for achieving specific and efficient expression of dual-color GECIs in astrocytes and neurons.

### 3.2 Simultaneous Recording of Astrocytic and Neuronal Ca^2+^ Activities is Achieved in Freely Behaving Mice

**Fig. 2:**
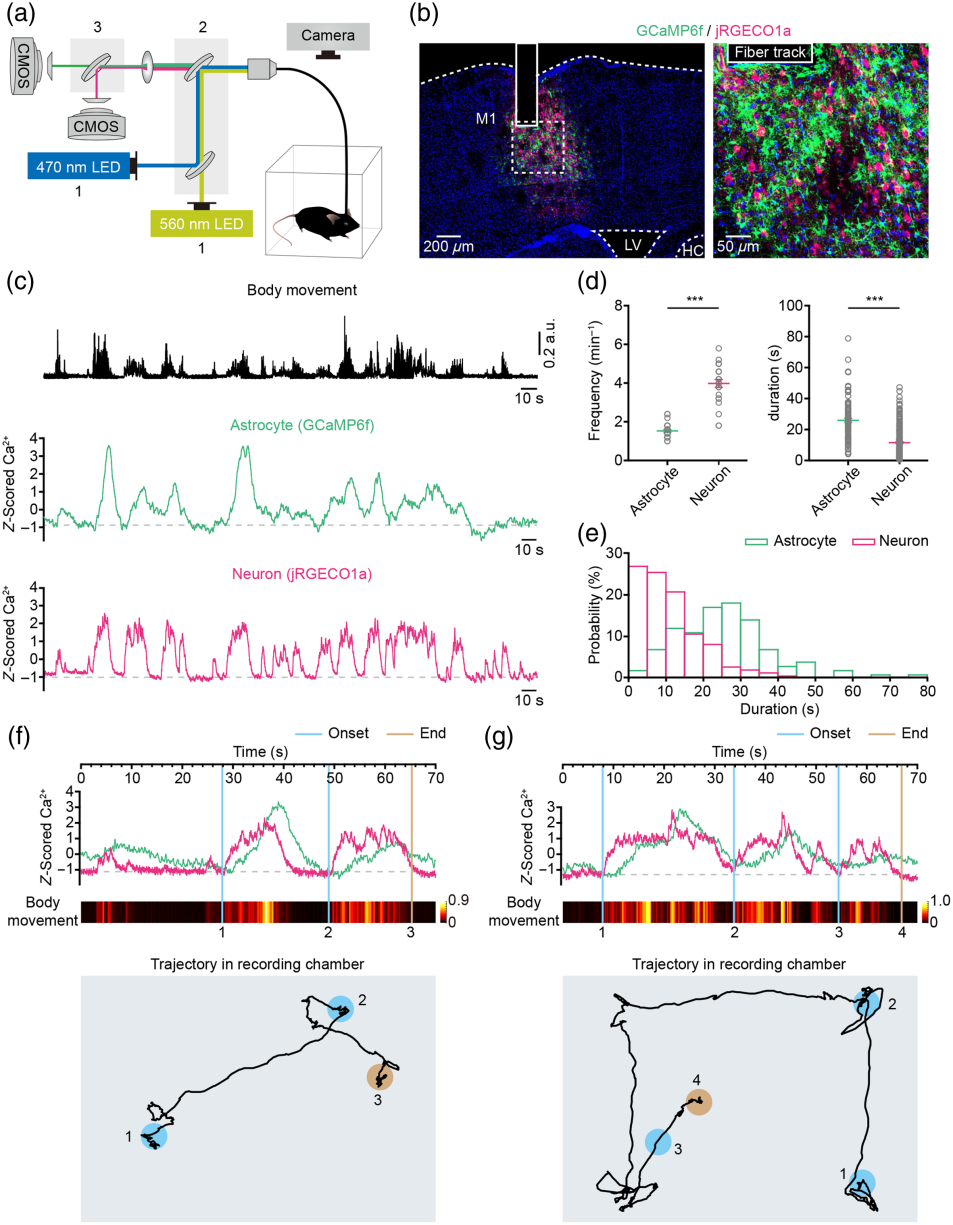
Simultaneous recording of astrocytic and neuronal ${\mathrm{Ca}}^{2+}$ transients alongside body movements in freely behaving mice. (a) Schematic illustration of the dual-color fiber photometry system used for simultaneous recording of astrocytic and neuronal ${\mathrm{Ca}}^{2+}$ transients in freely behaving mice. Excitation light (1), fluorescence cube with exchangeable filters (2), and the spectrometer (3) are shown. (b) Representative *post hoc* images (left, low power; right, high power) displaying astrocytes labeled with GCaMP6f (green) and neurons labeled with jRGECO1a (magenta) underneath the fiber tracks (column) in the primary motor cortex (M1). LV: lateral ventricle; HC: hippocampus. (c) Example showing body movements (black traces), simultaneously recorded astrocytic (green traces) and neuronal (magenta traces) ${\mathrm{Ca}}^{2+}$ transients following the virus injections. (d) Left, graphs summarizing the frequencies of astrocytic and neuronal ${\mathrm{Ca}}^{2+}$ transients in the cortex. n=25 trials from five mice (astrocytes versus neurons, Z=−5.8747, $P=4.24\times 10^{-09}$, ***P<0.001, Wilcoxon’ s rank-sum test). Right, graphs summarizing the durations of astrocytic and neuronal ${\mathrm{Ca}}^{2+}$ transients in the cortex. Astrocytes: n=99 trials from five mice, neurons: n=278 trials from five mice (astrocytes versus neurons, Z=−9.9920, $P=1.65\times 10^{-23}$, ***P<0.001, Wilcoxon’ s rank-sum test). Data are shown as mean ± s.e.m. (e) Distributions of the durations of astrocytic (green) and neuronal (magenta) ${\mathrm{Ca}}^{2+}$ transients in cortex. (f), (g) Trial-by-trial examples illustrating the correlations between astrocytic ${\mathrm{Ca}}^{2+}$ transients (green trace), neuronal ${\mathrm{Ca}}^{2+}$ transients (magenta trace), and body movements (color-coded intensity levels). Neuronal ${\mathrm{Ca}}^{2+}$ transient onset and end points are marked by blue and brown lines, respectively. At the bottom, the recording chambers are represented by rectangular gray boxes. Numbered blue and brown circles correspond to the upper lines, indicating the positions of neuronal ${\mathrm{Ca}}^{2+}$ transient onsets and endpoints. Black lines depict the trajectories of the mice.

The fiber photometry system comprises three main components: an excitation light source, a fluorescence cube with exchangeable filters, and a spectrometer [Fig. 2(a)]. To simultaneously measure green and red fluorophores, we employed a 470-nm LED and a 560-nm LED as excitation light sources, along with appropriate dual-band dichroic and emission filters. Each mouse was placed in a white, opaque, rectangular chamber 24 h after fiber probe implantation. During the fiber recording session, the behavior of freely behaving mice was recorded using a camera positioned above the chamber [Fig. 2(a)]. A fiber probe with a diameter of 200  μm was implanted above GCaMP6f-positive astrocytes and jRGECO1a-positive neurons [Fig. 2(b)], 3 weeks after virus injection (using ${\mathrm{AAV}5\text{-}\mathrm{GfaABC}}_{1}D\text{-}\text{cyto}$-GCaMP6f-SV40 and AAV9-hSyn-NES-jRGECO1a-SV40).

Using the device shown in Fig. 2(a), we recorded both body movements and the ${\mathrm{Ca}}^{2+}$ activities of astrocytes and neurons. Figure 2(c) presents a representative example of body movements (black traces) alongside simultaneously recorded astrocytic (green traces) and neuronal (magenta traces) ${\mathrm{Ca}}^{2+}$ activities during free exploration by mice in the open field. Z score normalization was utilized to represent the normalized magnitude, mitigating the inherent differences in the dynamic range of GCaMP6f and jRGECO1a. Statistical analysis indicated that astrocytic ${\mathrm{Ca}}^{2+}$ activities occur less frequently compared with neurons [Fig. 2(d), left], but have longer durations [Fig. 2(d), right]. Furthermore, most astrocytic ${\mathrm{Ca}}^{2+}$ activities last longer than 20 s, whereas neuronal ${\mathrm{Ca}}^{2+}$ activities primarily have durations shorter than 20 s [Fig. 2(e)].

To ascertain whether fluorescence signals from GCaMP6f or jRGECO1a recorded by the fiber photometry system faithfully report astrocytic or neuronal ${\mathrm{Ca}}^{2+}$ transients in freely behaving animals, we conducted *in vivo* recordings with GCaMP6f expressed in astrocytes and mCherry in neurons [Figs. S3(a) and S3(c) in the Supplementary Material], as well as with jRGECO1a in neurons and green fluorescent protein (GFP) in astrocytes [Figs. S3(e) and S3(g) in the Supplementary Material] within S1 and M1 regions. Our findings revealed frequent and transient increases in fluorescence signals exclusively in GCaMP6f and jRGECO1a, with no corresponding changes observed in mCherry and GFP fluorescence [Figs. S3(b), S3(d), S3(f), and S3(h) in the Supplementary Material]. This observation indicates that the fluorescence transients in GCaMP6f and jRGECO1a reflect ${\mathrm{Ca}}^{2+}$ influx associated with astrocytic or neuronal activity, rather than being artifacts of movement. Considering the heterogeneity of astrocytic ${\mathrm{Ca}}^{2+}$ transients, which include both microdomain and cell-wide scales,^4^ we next assessed the sensitivity of fiber photometry in detecting these transients. Previous studies have demonstrated that the majority of astrocytic ${\mathrm{Ca}}^{2+}$ transients in quiet awake mice are microdomain ${\mathrm{Ca}}^{2+}$ transients,^32^ whereas those observed in moving mice are primarily cell-wide “global” ${\mathrm{Ca}}^{2+}$ transients.^26^ Therefore, we utilized fiber photometry to record astrocytic ${\mathrm{Ca}}^{2+}$ transients in both stationary and moving mice. The results indicate that fiber photometry can effectively detect astrocytic ${\mathrm{Ca}}^{2+}$ transients under both conditions [Figs. S4(a)–S4(d) in the Supplementary Material]. The amplitude of astrocytic ${\mathrm{Ca}}^{2+}$ transients during movement states is approximately ten times greater than that observed in stationary states [Figs. S4(b) and S4(d) in the Supplementary Material]. Thus, the data validate its capability to identify both microdomain and cell-wide “global” astrocytic ${\mathrm{Ca}}^{2+}$ transients.

To investigate the role of astrocytic and neuronal ${\mathrm{Ca}}^{2+}$ activities in cognitive functions such as spatial exploration and memory, simultaneous recording of astrocytic and neuronal ${\mathrm{Ca}}^{2+}$ activity alongside mouse position in real time is essential. In this study, we concurrently recorded ${\mathrm{Ca}}^{2+}$ activities in astrocytes and neurons along with the movements of freely behaving mice [Fig. 2(c)]. The precise onset and cessation of neuronal ${\mathrm{Ca}}^{2+}$ activities could be clearly identified within the mouse trajectories as depicted in the simultaneously recorded videos [Figs. 2(f) and 2(g)]. Moreover, the real-time body movement status could be correlated with specific behavioral patterns of the mouse at these locations [Figs. 2(f) and 2(g)].

### 3.3 Movement Evokes Correlated Astrocytic and Neuronal Ca^2+^ Transients in Freely Behaving Mice

**Fig. 3:**
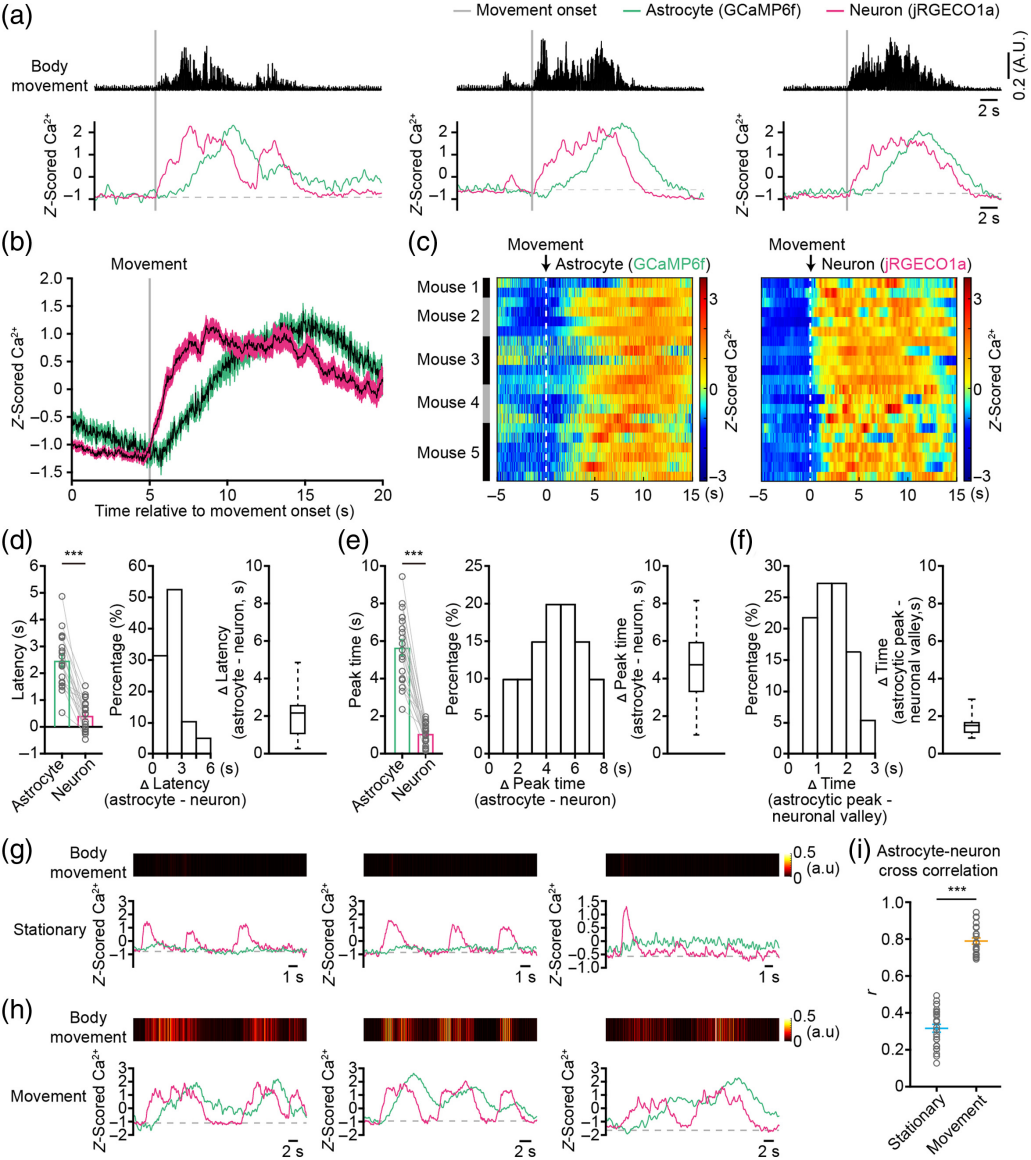
Correlated astrocytic and neuronal ${\mathrm{Ca}}^{2+}$ transients evoked by movement in freely behaving mice. (a) Representative astrocytic (green) and neuronal (magenta) ${\mathrm{Ca}}^{2+}$ transients simultaneously evoked by body movements (black). Movement onsets are depicted by gray bars. (b) Average astrocytic (green) and neuronal (magenta) ${\mathrm{Ca}}^{2+}$ transients evoked by movement (gray bar). n=21 trials from five mice. The shaded area represents SEM. (c) Color-coded intensities of astrocytic (left) and neuronal (right) ${\mathrm{Ca}}^{2+}$ transients evoked by movement (white dashed line) from different trials in the cortices. The trial mice for ${\mathrm{Ca}}^{2+}$ transients are indicated in the left black and gray columns. (d) Left, bar graphs summarizing the latencies of astrocytic and neuronal ${\mathrm{Ca}}^{2+}$ transients evoked by movements. n=19 trials from five mice (astrocytes versus neurons, Z=−3.8230, $P=1.32\times 10^{-04}$, ***P<0.001, two-sided Wilcoxon signed-rank test). Histogram (middle) and a bar graph (right) illustrating differences in latencies of astrocytic and neuronal ${\mathrm{Ca}}^{2+}$ transients evoked by movement. n=19 trials from five mice. (e) Left, bar graphs summarizing the peak times of astrocytic and neuronal ${\mathrm{Ca}}^{2+}$ transients evoked by movements. n=20 trials from five mice (astrocytes versus neurons, Z=−3.91993, $P=8.90\times 10^{-05}$, ***P<0.001, two-sided Wilcoxon signed-rank test). Histogram (middle) and a bar graph (right) illustrating differences in peak times of astrocytic and neuronal ${\mathrm{Ca}}^{2+}$ transients evoked by movement. n=20 trials from five mice. (f) Histogram (left) and a bar graph (right) illustrating differences between peak time of astrocytic ${\mathrm{Ca}}^{2+}$ transients and valley time of neuronal ${\mathrm{Ca}}^{2+}$ transients evoked by movement. n=20 trials from five mice. (g), (h) Representative examples illustrating the correlations between astrocytic ${\mathrm{Ca}}^{2+}$ transients (green trace), neuronal ${\mathrm{Ca}}^{2+}$ transients (magenta trace) and body movements (color-coded intensity levels) during stationary (g) and movement (h) states. (i) Mean cross-correlation r-value between astrocytic and neuronal ${\mathrm{Ca}}^{2+}$ transients during stationary and movement states. n=24 trials from five mice (stationary versus movement, Z=−5.9412, $P=2.83\times 10^{-09}$, ***P<0.001, Wilcoxon’ s rank-sum test). All data are shown as mean ± s.e.m.

Previous studies have demonstrated that movement can elicit ${\mathrm{Ca}}^{2+}$ transients not only in neurons^33^ but also in astrocytes.^20^^,^^26^^,^^29^ However, there is currently no suitable technique to investigate the correlation between movement-evoked ${\mathrm{Ca}}^{2+}$ transients in astrocytes and neurons during free behaviors. In this study, we utilized the dual-color fiber photometry system [Fig. 2(a)] to concurrently record ${\mathrm{Ca}}^{2+}$ transients in both astrocytes and neurons in the primary motor cortex (M1) of freely behaving mice. We then examined the dynamic relationship of movement-evoked ${\mathrm{Ca}}^{2+}$ transients in astrocytes and neurons. The results demonstrated that movement could trigger simultaneous ${\mathrm{Ca}}^{2+}$ transients in astrocytes and neurons [Fig. 3(a)]. Importantly, the temporal patterns of ${\mathrm{Ca}}^{2+}$ transients differed between astrocytes and neurons [Fig. 3(b)]. Specifically, movement-evoked astrocytic ${\mathrm{Ca}}^{2+}$ transients exhibited significantly longer latencies compared with those in neurons. In addition, the peak of astrocytic ${\mathrm{Ca}}^{2+}$ transients occurred later than that of neurons [Fig. 3(c)]. Statistical analysis indicated that the latency of movement-evoked ${\mathrm{Ca}}^{2+}$ transients was 2.47±0.24  s in astrocytes, compared with 0.42±0.13  s in neurons [Fig. 3(d), left]. This delay in astrocytic ${\mathrm{Ca}}^{2+}$ transients was ˜2.17s compared with neurons [Fig. 3(d), middle and right]. The peak time of movement-evoked astrocytic ${\mathrm{Ca}}^{2+}$ transients was 5.66±0.41  s in astrocytes and 1.07±0.12  s in neurons [Fig. 3(e), left].

Furthermore, the astrocytic peak time was delayed by about 4.73 s compared with neurons [Fig. 3(e), middle and right]. We found a close association between the continuous increase in astrocytic ${\mathrm{Ca}}^{2+}$ transients and the decrease in neuronal ${\mathrm{Ca}}^{2+}$ transients, resulting in the inter-locking of the astrocytic ${\mathrm{Ca}}^{2+}$ peak with the neuronal ${\mathrm{Ca}}^{2+}$ valley [Fig. 3(a)]. Statistical analysis indicates that the peak of astrocytic ${\mathrm{Ca}}^{2+}$ transients occurred ∼1.50  s before the valley of neuronal ${\mathrm{Ca}}^{2+}$ transients [Fig. 3(f), left and right]. The distinct temporal patterns of ${\mathrm{Ca}}^{2+}$ transients between astrocytes and neurons suggest unique mechanisms governing ${\mathrm{Ca}}^{2+}$ signaling in each cell type, as well as potential interactions between astrocytes and neurons.

Using *in vivo* two-photon ${\mathrm{Ca}}^{2+}$ imaging, a previous study suggested an increased correlation of astrocytic and neuronal ${\mathrm{Ca}}^{2+}$ transients during the movement state, compared with the stationary state.^29^ To further validate this observation in freely moving mice, we employed a fiber photometry system to record ${\mathrm{Ca}}^{2+}$ transients in astrocytes and neurons during both stationary and movement states. Subsequently, we evaluated the correlation between astrocytic and neuronal ${\mathrm{Ca}}^{2+}$ transients in these two states.

Our results demonstrated that in the stationary state, there was no synchronization between astrocytic and neuronal ${\mathrm{Ca}}^{2+}$ transients [Fig. 3(g)]. By contrast, during the movement state, astrocytic and neuronal ${\mathrm{Ca}}^{2+}$ transients fluctuated concurrently with the animal’s movements [Fig. 3(h)]. Statistical analysis revealed a significantly higher correlation between astrocytic and neuronal ${\mathrm{Ca}}^{2+}$ transients during the movement state (r=0.79±0.02) compared with the stationary state [r=0.32±0.02, Fig. 3(i)]. To further investigate the effects of neuronal activity on astrocytic ${\mathrm{Ca}}^{2+}$ transients, we employed random forest regression to predict astrocyte ${\mathrm{Ca}}^{2+}$ levels [Fig. S5(a) in the Supplementary Material]. This approach enabled us to account for approximately ∼29.66% of the variance in astrocyte ${\mathrm{Ca}}^{2+}$ during the stationary state and∼76.83% of the variance during the movement state [Fig. S5(a) in the Supplementary Material]. These results corroborate the earlier study conducted in head-fixed mice using *in vivo* two-photon ${\mathrm{Ca}}^{2+}$ imaging,^29^ indicating a more interconnected relationship between astrocytic and neuronal ${\mathrm{Ca}}^{2+}$ transients during movement than during stationary states.

### 3.4 Sensory Stimulation Induces Inter-Locked Astrocytic and Neuronal Ca^2+^ Transients in Freely Behaving Mice

**Fig. 4:**
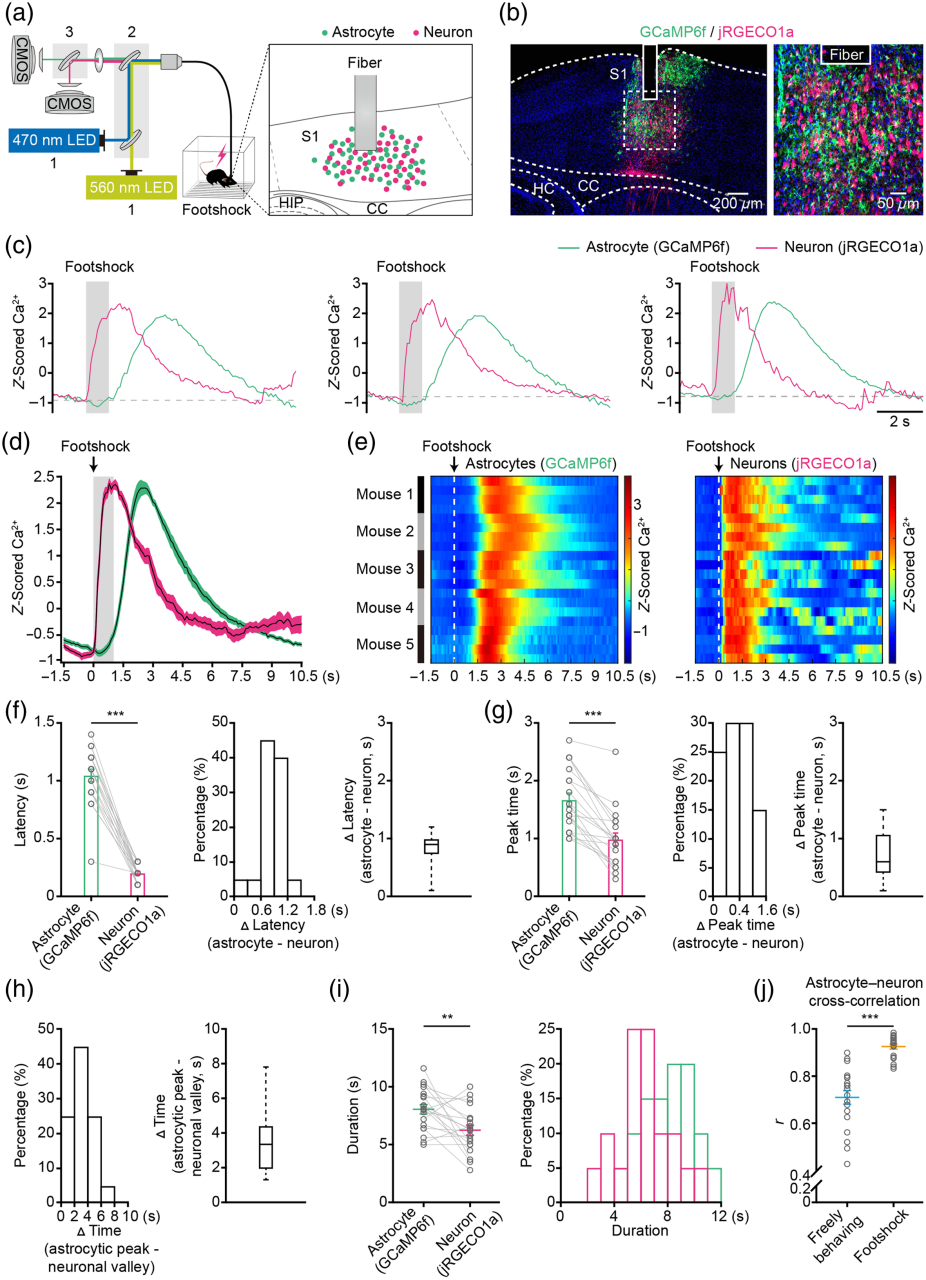
Inter-locked astrocytic and neuronal ${\mathrm{Ca}}^{2+}$ transients induced by footshock in freely behaving mice. (a) Schematic illustration of the fiber photometry system used for measuring dual-color GECIs expressed in astrocytes and neurons in the cortex during footshock stimuli. (b) Representative *post hoc* images (left, low power; right, high power) displaying astrocytes labeled with GCaMP6f (green) and neurons labeled with jRGECO1a (magenta) underneath the fiber tracks (column) in the primary somatosensory cortex (S1). CC: corpus callosum, HC: hippocampus. (c) Representative astrocytic (green) and neuronal (magenta) ${\mathrm{Ca}}^{2+}$ transients simultaneously induced by footshock (gray bar). (d) Average astrocytic (green) and neuronal (magenta) ${\mathrm{Ca}}^{2+}$ transients evoked by footshock (gray bar). n=20 trials from five mice. The shaded area represents SEM. (e) Color-coded intensities of astrocytic (left) and neuronal (right) ${\mathrm{Ca}}^{2+}$ transients induced by footshock (white dashed line) from different trials in the cortices. The trial mice for ${\mathrm{Ca}}^{2+}$ transients are indicated in the left black and gray columns. (f) Left, bar graphs summarizing the latencies of astrocytic and neuronal ${\mathrm{Ca}}^{2+}$ transients induced by footshock. n=20 trials from five mice (astrocytes versus neurons, Z=−3.9364, $P=8.30\times 10^{-05}$, ***P<0.001, two-sided Wilcoxon signed-rank test). Histogram (middle) and a bar graph (right) illustrating differences in latencies for astrocytic and neuronal ${\mathrm{Ca}}^{2+}$ transients induced by footshock. n=20 trials from five mice. (g) Left, bar graphs summarizing the peak times of astrocytic and neuronal ${\mathrm{Ca}}^{2+}$ transients induced by footshock. n=20 trials from five mice (astrocytes versus neurons, Z=−3.9244, $P=8.70\times 10^{-05}$, ***P<0.001, two-sided Wilcoxon signed-rank test). Histogram (middle) and a bar graph (right) illustrating differences in peak times of astrocytic and neuronal ${\mathrm{Ca}}^{2+}$ transients induced by footshock. n=20 trials from five mice. (h) Histogram (left) and a bar graph (right) illustrating differences between peak time of astrocytic ${\mathrm{Ca}}^{2+}$ transients and the valley of neuronal ${\mathrm{Ca}}^{2+}$ transients induced by footshock. n=20 trials from five mice. (i) Left, graphs summarizing the durations of astrocytic and neuronal ${\mathrm{Ca}}^{2+}$ transients induced by footshock. n=20 trials from five mice (astrocyte versus neuron, Z=−2.7183, $P=6.56\times 10^{-03}$, **P<0.01, two-sided Wilcoxon signed-rank test). Right, distributions of the durations of astrocytic (green) and neuronal (magenta) ${\mathrm{Ca}}^{2+}$ transients induced by footshock. (j) Mean cross-correlation r-value between astrocytic and neuronal ${\mathrm{Ca}}^{2+}$ transients during freely moving and footshock. n=20 trials from five mice (freely moving versus footshock, Z=−3.8646, $P=1.11\times 10^{-04}$, ***P<0.001, Wilcoxon’s rank-sum test). Data [(f) left, (g) left, (i) left, and (j)] are shown as mean ± s.e.m.

Astrocytes demonstrate stimulus-dependent responses to sensory input,^7^ influencing neuronal network activity and modulating the overall dynamic range of cortical network activity.^7^^,^^34^ However, it remains unclear whether and how astrocytic and neuronal ${\mathrm{Ca}}^{2+}$ transients are correlated in response to sensory stimuli in freely behaving animals. By employing a two-channel fiber photometry system [Fig. 4(a)] and utilizing two different GECIs (jRGECO1a and GCaMP6f) in the primary somatosensory cortex (S1) [Fig. 4(b)], we investigated these questions while delivering electrical stimuli to the hind paws of freely behaving mice. This approach allowed precise stimulation of sensory inputs in a controlled manner [Fig. 4(a)]. As depicted in Figs. 1(c) and 1(d), astrocytes were labeled with GCaMP6f and neurons with jRGECO1a, followed by placement of the fiber photometry probe above these GECI-labeled cells [Fig. 4(b)].

Our study demonstrates that astrocytes and neurons exhibit concurrent responses to sensory stimuli in S1 [Fig. 4(c)]. In addition, we observed an inter-locked relationship between neuronal and astrocytic ${\mathrm{Ca}}^{2+}$ transients induced by sensory stimuli, revealing a hysteresis pattern indicative of a negative feedback loop [Fig. 4(d)]. We further analyzed the temporal patterns of astrocytic and neuronal ${\mathrm{Ca}}^{2+}$ transients triggered by sensory stimuli. Similar to the findings with movement-evoked ${\mathrm{Ca}}^{2+}$ transients [Fig. 3(c)], our results indicated that sensory stimuli-induced astrocytic ${\mathrm{Ca}}^{2+}$ transients have significantly longer latencies compared with neurons.

Furthermore, we observed that the peak of astrocytic ${\mathrm{Ca}}^{2+}$ transients occurred later than that of neurons [Fig. 4(e)]. Statistical analysis revealed that the latency of sensory stimuli-induced ${\mathrm{Ca}}^{2+}$ transients in astrocytes was 1.05±0.05  s, whereas it was 0.20±0.01  s in neurons [Fig. 4(f), left]. Specifically, the latency of astrocytic ${\mathrm{Ca}}^{2+}$ transients was delayed by ∼0.90  s compared with neurons [Fig. 4(f), middle and right]. The peak time of sensory stimuli-induced ${\mathrm{Ca}}^{2+}$ transients was 1.67±0.12  s in astrocytes and 0.99±0.11  s in neurons [Fig. 4(g), left]. In addition, the astrocytic peak time was delayed by about 0.60 s compared with neurons [Fig. 4(g), middle and right].

We also noted that the increase in astrocytic ${\mathrm{Ca}}^{2+}$ transients coincides with the decrease in neuronal ${\mathrm{Ca}}^{2+}$ transients [Fig. 4(d)]. Statistical analysis indicated that the peak of astrocytic ${\mathrm{Ca}}^{2+}$ transients preceded the valley of neuronal ${\mathrm{Ca}}^{2+}$ transients by ∼3.35  s [Fig. 4(h) left and right]. This pattern exhibited hysteresis, suggesting a potential negative regulatory mechanism between astrocytic and neuronal ${\mathrm{Ca}}^{2+}$ signaling. In addition, the duration of sensory stimuli-induced ${\mathrm{Ca}}^{2+}$ transients was 8.05±0.41  s in astrocytes, which was notably longer than in neurons (6.24±0.44  s) [Fig. 4(i), left and right].

Next, we evaluated the correlation between astrocytic and neuronal ${\mathrm{Ca}}^{2+}$ transients both during free behavior and sensory stimulation (footshok). Statistical analysis demonstrated a significantly higher correlation between astrocytic and neuronal ${\mathrm{Ca}}^{2+}$ transients during sensory stimuli (r=0.93±0.01) compared with freely behaving conditions (r=0.71±0.03) [Fig. 4(j)]. To gain a better understanding of the impact of neuronal activity on astrocytic ${\mathrm{Ca}}^{2+}$ transients, we utilized random forest regression to forecast the levels of ${\mathrm{Ca}}^{2+}$ in astrocytes [Fig. S5(c) in the Supplementary Material]. This methodology allowed us to explain around ∼48.58% of the variance in astrocyte ${\mathrm{Ca}}^{2+}$ during free behavior and about ∼69.38% of the variance in response to sensory stimuli [Fig. S5(c) in the Supplementary Material]. These results indicate a more interconnected relationship between astrocytic and neuronal ${\mathrm{Ca}}^{2+}$ transients during sensory stimuli than during free behavior.

### 3.5 Multi-Regional Measurement of Astrocytic and Neuronal Ca^2+^ Transients Reveals the Movement-Enhanced Astrocyte-Neuronal Networks on a Brain-Wide Scale

**Fig. 5:**
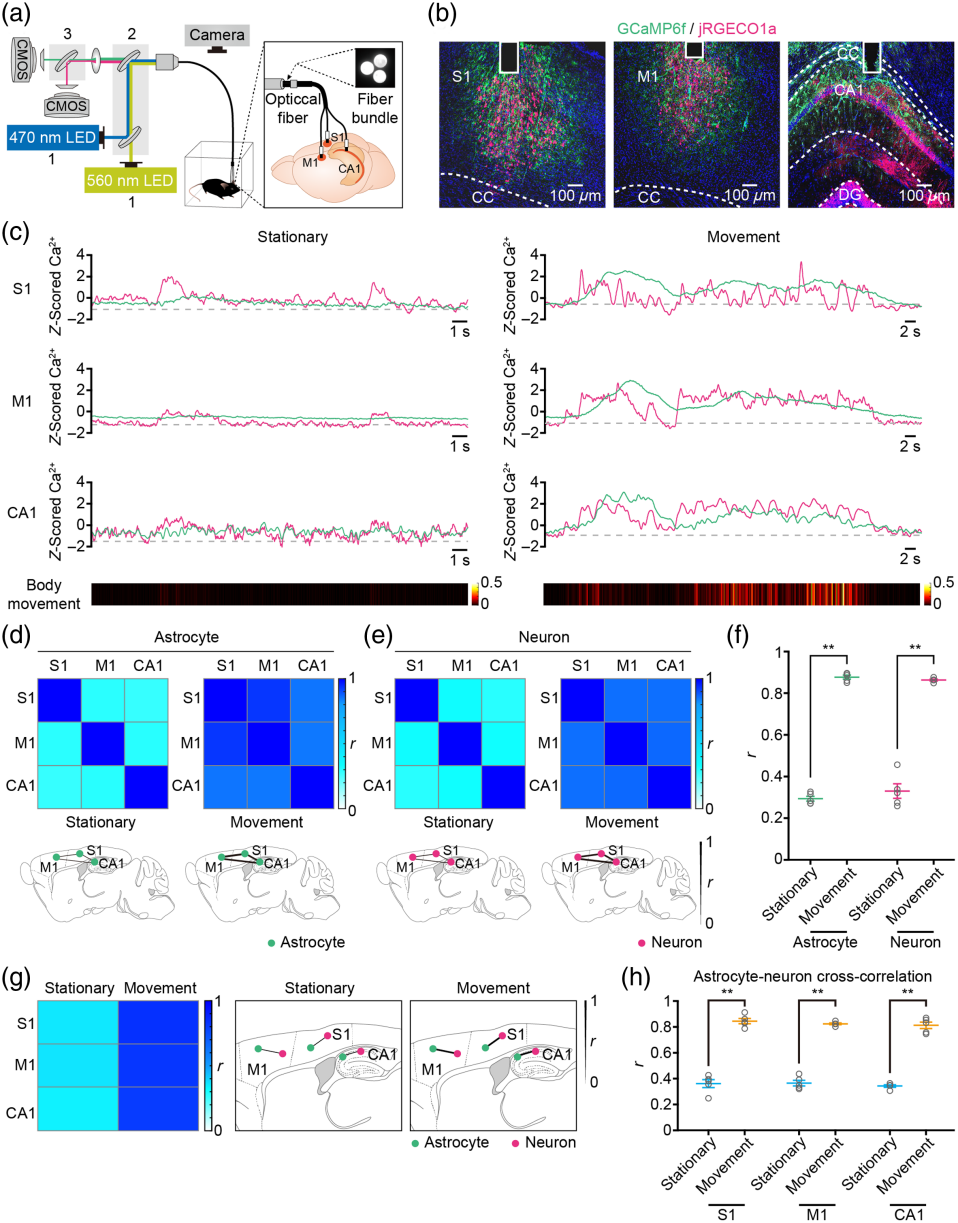
Simultaneous measurement of astrocytic and neuronal ${\mathrm{Ca}}^{2+}$ transients at multiple brain regions in freely behaving mice. (a) Schematic illustration of the dual-color multi-fiber photometry system used for simultaneously recording astrocytic and neuronal ${\mathrm{Ca}}^{2+}$ transients in the primary somatosensory cortex (S1), the primary motor cortex (M1), and the hippocampal CA1 region (CA1) of freely behaving mice. (b) Representative *post hoc* images displaying astrocytes labeled with GCaMP6f (green) and neurons labeled with jRGECO1a (magenta) underneath the fiber track (column) in the S1 (left), M1 (middle), and CA1. S1: primary somatosensory cortex, M1: primary motor cortex, CA1: hippocampal CA1 region, CC: corpus callosum, DG: dentate gyrus. (c) Representative astrocytic (green) and neuronal (magenta) ${\mathrm{Ca}}^{2+}$ transients simultaneously recorded in S1, M1, and CA1 during stationary (left) and movement (right) states. Body movement (bottom, color-coded intensity levels) is also simultaneously recorded. (d), (e) Top, heat maps of the Spearman correlation coefficient (r) of astrocytic (d) or neuronal (e) ${\mathrm{Ca}}^{2+}$ transients between brain regions represented in panel (c) during stationary and movement states. Bottom, spatial representations of r values of astrocytic (d) or neuronal (e) ${\mathrm{Ca}}^{2+}$ transients between different brain regions during stationary or movement states. (f) Mean r-value of astrocytic or neuronal ${\mathrm{Ca}}^{2+}$ transients between all brain regions during stationary or movement states. n=60 trials from five mice (stationary versus movement: astrocytes: Z=−2.6271, $P=8.61\times 10^{-03}$, **P<0.01; neurons: Z=−2.6434, $P=8.21\times 10^{-03}$, **P<0.01; Wilcoxon’s rank-sum test). (g) Left, heat maps of the cross-correlation coefficient (r) calculated between astrocytic and neuronal ${\mathrm{Ca}}^{2+}$ transients in brain regions presented in panel (c) during stationary and movement states. Right, spatial representations of r-values between astrocytic and neuronal ${\mathrm{Ca}}^{2+}$ transients in different brain regions during stationary and movement states. (h) Mean r-value between the astrocytic and neuronal ${\mathrm{Ca}}^{2+}$ transients during stationary or movement in S1, M1, and CA1. n=20 trials from five mice (stationary versus movement: S1: Z=−2.6112, $P=9.02\times 10^{-03}$, **P<0.01; M1: Z=−2.6434, $P=8.21\times 10^{-03}$, **P<0.01; CA1: Z=−2.6434, $P=8.21\times 10^{-03}$, **P<0.01; Wilcoxon’s rank-sum test). Data (f) and (h) are shown as mean ± s.e.m.

Real-time measurement of astrocyte-neuronal network signals from multiple brain regions is crucial for understanding how circuit activity patterns influence behavior. However, current techniques for simultaneously assessing these dynamics in behaving animals remain limited. In this study, we developed an optical setup for parallel fluorescence recordings using multi-fiber photometry [Fig. 5(a)] and employed cell-specific dual-color GECIs [GCaMP6f and jRGECO1a, Fig. 5(b)]. This setup enabled simultaneous recording of astrocytic and neuronal ${\mathrm{Ca}}^{2+}$ transients from multiple brain regions in freely behaving mice.

The optical setup comprised a three-fiber patchcord, tightly bundled at one end and branching into three at the other [Fig. 5(a)]. This setup served to both deliver excitation and collect emitted light. Each fiber branch is connected to a fiber optic interface inserted into distinct brain regions. Signal recording utilized two CMOS cameras, capturing two fluorescence emissions simultaneously from all three fibers at the bundled end [Fig. 5(a)].

For cell-specific ${\mathrm{Ca}}^{2+}$ transients, astrocytes were labeled with GCaMP6f and neurons with jRGECO1a. Subsequently, we surgically implanted three optical fibers to target three specific brain regions: S1, M1, and CA1 regions [Fig. 5(b)]. These regions were previously virally injected with GCaMP6f and jRGECO1a, and *post hoc* histology confirmed the positions of fiber tips and GECI labeling [Fig. 5(b)]. We then conducted simultaneous recordings of astrocytic and neuronal ${\mathrm{Ca}}^{2+}$ transients across these brain regions using all three optical fibers in freely behaving mice. Robust ${\mathrm{Ca}}^{2+}$ transients were observed in both astrocytes and neurons during stationary and movement states [Fig. 5(c)]. Importantly, during movement states, we observed increased synchronization of astrocytic and neuronal ${\mathrm{Ca}}^{2+}$ transients compared with stationary states [Fig. 5(c)]. These findings suggest a potential relationship between movement and the coordination of astrocytic and neuronal ${\mathrm{Ca}}^{2+}$ transients across distinct brain regions.

Multi-fiber photometry facilitates the reconstruction of astrocyte-neuronal networks in the brain associated with behavior. We assessed brain-wide functional connectivity by calculating the Spearman correlation coefficient (r) of astrocytic and neuronal ${\mathrm{Ca}}^{2+}$ transients between different regions during stationary and movement states [Figs. 5(d) and 5(e), top panels]. Connections between brain regions were represented with varying link thicknesses based on correlation strength [Figs. 5(d) and 5(e), bottom panels]. Notably, thicker links were observed in the astrocytic and neuronal networks during movement states compared with stationary states [Figs. 5(d) and 5(e), bottom panels]. Statistical analysis revealed significantly higher correlation coefficients of astrocytic or neuronal ${\mathrm{Ca}}^{2+}$ transients between brain regions during movement states (astrocyte: r=0.88±0.01; neuron: r=0.86±0.005) compared with stationary states [astrocyte: r=0.29±0.01; neuron: r=0.33±0.03, Fig. 5(f)].

In addition, to explore the relationship between movement-induced ${\mathrm{Ca}}^{2+}$ transients in astrocytes and nearby neurons, cross-correlations between astrocytic and neuronal ${\mathrm{Ca}}^{2+}$ transients across different brain regions during stationary and movement states were analyzed. Results demonstrated a pronounced increase in cross-correlations during movement states (S1: r=0.85±0.02; M1: r=0.82±0.01; CA1: r=0.81±0.03) compared with stationary states [S1: r=0.36±0.03; M1: r=0.37±0.02; CA1: r=0.34±0.01, Figs. 5(g) and 5(h)]. Moreover, we employed neuronal activity data to predict astrocytic ${\mathrm{Ca}}^{2+}$ transients using random forest regression. The results indicate that the predictable percentages of variance in astrocyte ${\mathrm{Ca}}^{2+}$ levels are elevated across various brain regions during the movement state [Fig. S5(b) in the Supplementary Material, S1: ∼46.71%; M1: ∼68.44%; CA1: ∼70.12%] compared with the stationary state [Fig. S5(b) in the Supplementary Material, S1: ∼41.09%; M1: ∼6.11%; CA1: ∼48.44%]. Thus, this method provides deeper insights into the interactions between astrocytes and neurons across and within brain regions during different behavioral states.

### 3.6 Multi-regional Measurement of Astrocytic and Neuronal ${\mathrm{Ca}}^{2+}$ Transients Illuminates Heightened Functional Brain-Wide Astrocyte-Neuronal Networks during Sensory Stimuli

**Fig. 6:**
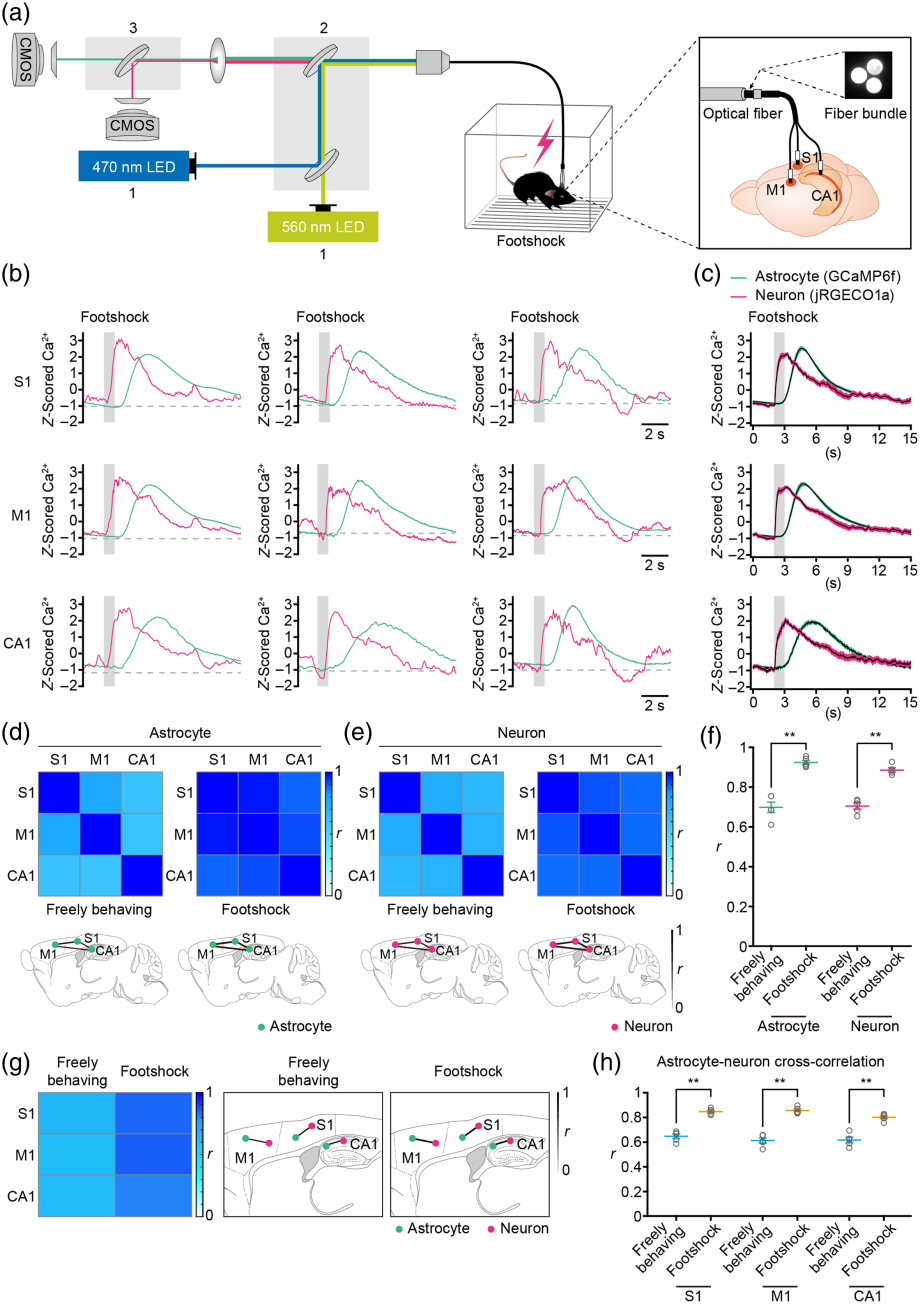
Simultaneous measurement of astrocytic and neuronal ${\mathrm{Ca}}^{2+}$ transients induced by sensory stimuli at multiple brain regions. (a) Schematic illustration of the dual-color multi-fiber photometry system used for simultaneously recording astrocytic and neuronal ${\mathrm{Ca}}^{2+}$ transients in the primary somatosensory cortex (S1), the primary motor cortex (M1), and hippocampal CA1 region (CA1) during footshock. (b) Representative astrocytic (green) and neuronal (magenta) ${\mathrm{Ca}}^{2+}$ transients simultaneously recorded in S1, M1, and CA1 during footshock (gray bar). (c) Average astrocytic (green) and neuronal (magenta) ${\mathrm{Ca}}^{2+}$ transients simultaneously recorded in S1, M1, and CA1 during footshock (grey bar). n=20 trials from five mice. The shaded area represents SEM. (d), (e) Top, heat maps of the Spearman correlation coefficient (r) of astrocytic (d) or neuronal (e) ${\mathrm{Ca}}^{2+}$ transients between brain regions represented in panels (b) and (c) during freely behaving and footshock. Bottom, spatial representations of r values of astrocytic (d) or neuronal (e) ${\mathrm{Ca}}^{2+}$ transients between different brain regions during freely behaving and footshock. (f) Mean r-value of astrocytic or neuronal ${\mathrm{Ca}}^{2+}$ transients between all brain regions during freely behaving or footshock. n=20 trials from five mice (freely behaving versus footshock, astrocytes: Z=−2.6352, $P=8.41\times 10^{-03}$, **P<0.01; neurons: Z=−2.6112, $P=9.02\times 10^{-03}$, **P<0.01; Wilcoxon’s rank-sum test). (g) Left, heat maps of cross-correlation coefficient (r) calculated between astrocytic and neuronal ${\mathrm{Ca}}^{2+}$ transients in brain regions in panels (b) and (c) during freely behaving and footshock. Right, spatial representations of r-values between astrocytic and neuronal ${\mathrm{Ca}}^{2+}$ transients in different brain regions during freely behaving and footshock. (h) Mean r-value between the astrocytic and neuronal ${\mathrm{Ca}}^{2+}$ transients during freely behaving and footshock in S1, M1, and CA1. n=20 trials from five mice (freely behaving versus footshock: S1: Z=−2.6191, $P=8.82\times 10^{-03}$, **P<0.001; M1: Z=2.6112, $P=9.02\times 10^{-03}$, **P<0.01; CA1: Z=−2.6112, $P=9.02\times 10^{-03}$, **P<0.01; Wilcoxon’s rank-sum test). Data (f) and (h) are shown as mean ± s.e.m.

To explore astrocytic and neuronal responses to sensory stimulation at a whole-brain level, we employed dual-color multi-fiber photometry techniques to monitor ${\mathrm{Ca}}^{2+}$ transients in astrocytes and neurons across brain-wide networks [Fig. 6(a)]. In this investigation, we simultaneously recorded astrocytic and neuronal ${\mathrm{Ca}}^{2+}$ transients in response to footshock in three distinct brain regions: S1, M1, and CA1 region [Fig. 6(a)]. Our results reveal consistent and synchronized ${\mathrm{Ca}}^{2+}$ transients in both astrocytes and neurons elicited by sensory stimuli (footshock) across these brain regions [Figs. 6(b) and 6(c)].

To evaluate brain-wide functional connectivity in response to sensory stimulation, we computed Spearman correlation coefficients (r) of astrocytic and neuronal ${\mathrm{Ca}}^{2+}$ transients between different brain regions during both freely behaving and footshock stimulation [Figs. 6(d) and 6(e), top panels]. The strength of these correlations was depicted by varying link thicknesses connecting the brain regions in our analysis [Figs. 6(d) and 6(e), bottom panels]. This method enabled visualization and quantitative assessment of astrocytic and neuronal functional connectivity across brain regions in response to sensory stimuli. Notably, we observed increased connectivity in both astrocytic and neuronal networks during footshock stimulation compared with freely behaving conditions [Figs. 6(d) and 6(e), bottom panels]. Statistical analysis demonstrated a significant rise in the correlation coefficients of astrocytic and neuronal ${\mathrm{Ca}}^{2+}$ transients across brain regions during footshock stimuli (astrocyte: r=0.92±0.01; neuron: r=0.89±0.01) compared with freely behaving [astrocyte: r=0.70±0.03; neuron: r=0.70±0.02; Fig. 6(f)].

In addition, we investigated cross-correlations between astrocytic and neuronal ${\mathrm{Ca}}^{2+}$ transients in various brain regions during both freely behaving and footshock stimuli to explore the relationship between sensory stimulation-induced ${\mathrm{Ca}}^{2+}$ dynamics. Our findings revealed a significant increase in cross-correlations across all brain regions during footshock stimulation (S1: r=0.85±0.01; M1: r=0.86±0.001; CA1: r=0.80±0.01) compared with freely behaving conditions [S1: r=0.65±0.02; M1: r=0.61±0.02; CA1: r=0.62±0.02; Figs. 6(g) and 6(h)]. Moreover, we utilized neuronal activity data to predict astrocytic ${\mathrm{Ca}}^{2+}$ transients through random forest regression. The results demonstrate that the percentage of variance in astrocyte ${\mathrm{Ca}}^{2+}$ levels that can be predicted is significantly higher across various brain regions during sensory stimuli [Fig. S5(d) in the Supplementary Material, S1: ∼79.68%; M1: ∼83.30%; CA1: ∼69.30%] in comparison to free behavior [Fig. S5(b) in the Supplementary Material; S1: ∼56.60%; M1: ∼61.35%; CA1: ∼37.90%]. Thus, this approach provides comprehensive insights into the dynamics of astrocyte-neuronal networks in response to sensory stimulation across multiple brain areas.

## 4 Discussion

In this study, we integrated cell-specific dual-color GECIs and fiber photometry to concurrently capture astrocytic and neuronal ${\mathrm{Ca}}^{2+}$ transients in freely behaving mice. This innovative approach enables the examination of astrocyte-neuronal networks across various behavioral patterns, shedding light on their functional dynamics. Moreover, by utilizing multi-fiber photometry and cell-specific dual-color GECIs, we achieved real-time recording of astrocytic and neuronal ${\mathrm{Ca}}^{2+}$ transients across multiple brain regions. This methodology is crucial for deciphering the intricate interactions between astrocytes and neurons within and across different brain regions in the context of specific behaviors. Furthermore, it facilitates the exploration of the roles played by astrocyte-neuronal networks at a comprehensive, whole-brain level.

Known as the “astrocyte-neuronal network,” the two-way communication between astrocytes and neurons plays a crucial role in various behaviors and brain functions.^6^^,^^7^ Astrocytes, furthermore, detect and integrate arousal states and sensory inputs through changes in intracellular ${\mathrm{Ca}}^{2+}$, which are essential for optimizing behavior.^35^ Several techniques are currently employed to simultaneously monitor astrocytic and neuronal activities, including *in vivo* two-photon ${\mathrm{Ca}}^{2+}$ imaging,^29^ the combination of two-photon ${\mathrm{Ca}}^{2+}$ imaging with electrophysiological recording,^6^ and fiber photometry.^21^ However, it remains unclear whether there is an optimal method to accurately record the ${\mathrm{Ca}}^{2+}$ responses of astrocyte-neuronal networks to specific behaviors. In this study, we utilized dual-color fiber photometry to simultaneously track astrocytic and neuronal ${\mathrm{Ca}}^{2+}$ transients during spontaneous movement (Fig. 3) and footshock stimulation (Fig. 4). The findings reveal synchronized responses from both astrocytes and neurons to these stimuli. Notably, the temporal patterns of astrocytic and neuronal ${\mathrm{Ca}}^{2+}$ transients exhibit a hysteresis pattern, indicating a bidirectional interaction where neurons initially facilitate astrocytes and subsequently astrocytes inhibit neurons. This observation aligns with previous studies utilizing electrophysiology^34^ and two-photon ${\mathrm{Ca}}^{2+}$ imaging techniques.^7^^,^^29^ Our study offers real-time and precise quantification of ${\mathrm{Ca}}^{2+}$ responses in astrocyte-neuronal networks in freely behaving mice, providing insights into the roles of this network in responding to specific behaviors.

The adult brain consists of multiple regions, each characterized by its unique combination of neuronal subtypes and distinct astrocytes.^12^ This suggests potential regional specialization within the astrocyte-neuronal network. Furthermore, behaviors critically rely on synchronized activity across multiple brain regions, where both neurons and astrocytes operate concurrently and in parallel during various behavioral contexts.^3^^,^^17^^,^^18^ It is established that astrocytic and neuronal ${\mathrm{Ca}}^{2+}$ transients are influenced by and adapted to activity patterns within local neuronal circuits.^35^ Thus, simultaneous recording of astrocytic and neuronal ${\mathrm{Ca}}^{2+}$ transients across multiple brain regions provides a valuable metric for evaluating the diversity and coordination of the astrocyte-neuronal network at a functional level. Yang and colleagues have introduced the multiarea two-photon real-time *in vivo* explorer, which enables the investigation of multiarea neuronal ${\mathrm{Ca}}^{2+}$ transients in head-fixed mice with single-cell resolution.^36^ Furthermore, researchers can record ${\mathrm{Ca}}^{2+}$ transients from multiple brain regions simultaneously in freely behaving mice,^23^^,^^25^ using either frame-projected independent-fiber photometry or arrays of optical fibers. However, there remains a lack of suitable methods for concurrently recording astrocytic and neuronal ${\mathrm{Ca}}^{2+}$ transients across multiple brain regions. In this study, we have successfully demonstrated the capability of combining multi-fiber photometry with cell-type specific dual-color GECIs to monitor ${\mathrm{Ca}}^{2+}$ transients in both astrocytes and neurons in freely behaving mice across multiple brain regions. Importantly, this methodology is not limited to mice but can also be effectively applied to other mammalian species, including rats and non-human primates.^37^ Previous research^24^ and our current findings (Fig. 2) support the feasibility of utilizing multiple indicator systems simultaneously through multi-color approaches. Therefore, the full potential of multi-fiber photometry with multi-color techniques can be realized by integrating multiple GECIs for specific labeling of cell types or pathways. This advancement allows researchers to monitor the activities of different cell types or pathways in various brain regions simultaneously.

Behavioral patterns in animals arise from neuronal activity that is distributed across brain networks.^25^ Specifically, many perceptual, cognitive, and motor functions rely not only on the neuronal activity spread throughout various regions of the brain but also on the effective communication of this activity between these areas. Consequently, to understand the neural substrates of complex behaviors, it is essential to comprehend the factors that regulate signal transmission across multiple brain regions and how this communication is orchestrated to facilitate adaptable behavior.^18^^,^^38^^,^^39^ Temporal coordination serves as a crucial indicator of the robustness of functional connectivity within neural networks.^18^ Neurons tend to respond more robustly to synchronous inputs from presynaptic neurons rather than asynchronous inputs, as they integrate synaptic inputs within defined time windows.^40^^–^^42^ In addition, the coherence of activity serves as a pivotal metric for assessing communication between different brain regions, as optimal inter-regional communication occurs when activity phases are well-synchronized.^18^^,^^43^ In this study, we have observed that spontaneous movement and sensory stimulation induce synchronous and coherent activity within astrocytic and neuronal networks across multiple brain regions, including S1, M1, and CA1 [Figs. 5(d)–5(f) and 6(d)–6(f)]. This indicates heightened astrocyte-neuronal communication between different brain regions during arousal states and sensory inputs. The underlying mechanism involves astrocytes in various brain regions sensing transient arousal states through neuromodulator release and responding to sensory inputs via local synaptic activity.^35^ This leads to concurrent increases in astrocytic ${\mathrm{Ca}}^{2+}$ transients that propagate^44^ and result in synchronized and coherent neuronal activities across diverse brain regions. Furthermore, subtle variations in astrocytic and neuronal activity patterns are evident among different brain regions. Specifically, astrocytic and neuronal ${\mathrm{Ca}}^{2+}$ transients in S1 exhibit closer coordination with those in M1 compared with CA1 (Figs. 5 and 6). This distinction may stem from a greater similarity in astrocytic and neuronal properties between S1 and M1, contrasting with CA1.^12^

The combination of multi-fiber photometry with cell-specific dual-color GECIs has facilitated the simultaneous recording of astrocytic and neuronal ${\mathrm{Ca}}^{2+}$ transients across diverse brain regions in freely behaving mice. By applying this technique, we can explore deeper insights into brain-wide astrocyte-neuronal networks in freely behaving animals, an area that has previously been inaccessible to investigation. First, researchers can examine alterations in the functional connectivity patterns of astrocyte-neuronal networks across multiple brain regions during various behaviors and distinct external stimuli. Accumulating experimental evidence suggests that diverse behaviors arise from the coordinated activity of astrocyte-neuronal networks across multiple brain regions.^2^^,^^3^^,^^8^ However, it remains uncertain how astrocyte-neuronal networks across different regions coordinate to respond to changes in behavior or sensory stimuli. In the present study, we found that the functional connectivity of astrocyte-neuronal networks across multiple brain regions increased during movement states or sensory stimulation compared with stationary states or freely behaving conditions [Figs. 5(d)–5(f) and 6(d)–6(f)]. This indicates that animals adopt optimized patterns of brain-wide astrocyte-neuronal network functional connectivity to modulate behaviors or respond to external stimuli. Second, using this method, region-specific changes within astrocyte-neuronal networks can be detected. Neurons and astrocytes in the brain exhibit diverse properties depending on their respective regions.^12^ Nonetheless, the question of whether and how astrocyte-neuronal networks employ region-specific mechanisms for the regulation of distinct neural circuits is not fully understood. In our present investigation, we found that changes in astrocyte-neuronal network activities were more similar between S1 and M1 compared with CA1 [Figs. 5(d)–5(f) and 6(d)–6(f)]. This suggests that brain regions composed of similar astrocytic and neuronal properties are likely to exhibit more comparable responses to changes in intrinsic states or external stimuli. Finally, the interactions between astrocytes and neurons, both across and within various brain regions, can be investigated in the context of different behavioral states and external stimuli. It is well established that astrocytes and neurons can communicate bidirectionally at the tripartite synapse.^1^ On the one hand, astrocytes sense neuronal network activity by responding with ${\mathrm{Ca}}^{2+}$ activity to neural signals in vivo; on the other hand, astrocyte activation or manipulation modulates neural network activity.^1^^,^^3^ However, how interaction patterns between astrocytes and neurons are modified in response to various behaviors or external stimuli remains an unresolved issue. Our study demonstrated a significant increase in cross-correlations between astrocytic and neuronal ${\mathrm{Ca}}^{2+}$ transients during movement [Figs. 5(g) and 5(h)] and footshock stimulation [Figs. 6(g) and 6(h)] across all brain regions. These findings indicate that communication between astrocytes and neurons is heightened by body locomotion and footshock, suggesting that bidirectional astrocyte-neuron communication can be modulated by both instinctual arousal states and external stimuli.

Looking forward, significant advancements are anticipated in several critical areas. First, further research is required to expand the repertoire of genetically encoded indicators (GEIs) to encompass various biological molecules, particularly neurotransmitters and neuropeptides.^45^^,^^46^ Coupled with these GEIs, observing direct interactions between astrocytes and neurons will provide valuable insights into the physiological mechanisms of astrocyte-neuronal networks. Moreover, high-density multi-fiber arrays have proven invaluable for studying large-scale brain dynamics during behaviors.^25^ Utilizing such arrays enables researchers to monitor and analyze activities within astrocyte-neuronal networks across numerous brain regions in mammals, covering 12 to 48 regions.^25^ This advanced technology facilitates a comprehensive understanding of the intricate astrocyte-neuronal networks and the transmission of signals across brain regions. Finally, integrating other modalities such as electrophysiology^47^^,^^48^ is crucial to overcome the limitations of photometry technology in studying brain signals. Combining these techniques allows researchers to complement and augment data obtained from photometry, thereby enhancing our understanding of neural activity on a broader scale.

One limitation of this method that should be noted is the potential formation of glial scars resulting from fiber photometry implantation. Increased reactive astrogliosis and a compromised immune microenvironment are the primary factors contributing to the development of glial scars.^49^^,^^50^ To mitigate their formation, we employed several strategies: (1) we controlled the insertion speed during fiber implantation to a maximum of 10  μm/s to minimize tissue damage that could lead to reactive astrogliosis, (2) we ensured the sterilization of fiber photometry and surgical instruments and promptly addressed any tissue bleeding during surgery to improve the immune microenvironment following fiber implantation, and (3) we recorded astrocytic ${\mathrm{Ca}}^{2+}$ transients over a period of 21 days following fiber photometry implantation, allowing sufficient time for recovery from the associated injury. Through these methods, we effectively reduced the formation of glial scars during the surgery.

We anticipate that the combination of multi-fiber photometry with cell-specific dual-color GEIs will be crucial for examining the intricate functional structure of astrocyte-neuronal networks. This technique not only enables the investigation of bidirectional communication between neurons and astrocytes but also facilitates the reconstruction of functional networks associated with specific behaviors. Furthermore, by employing advanced network analysis techniques, data obtained from this technique should contribute significantly to mapping multi-regional functional connectivity of astrocyte-neuronal networks during behavioral tasks. The diversity and complexity of astrocyte-neuron signaling provide a high degree of flexibility to the networks that underlie brain function.

## Supplementary Material



## Data Availability

The data supporting the results presented in this paper are available from the corresponding author upon reasonable request.
