# Supplementary material for: Mapping multi-regional functional connectivity of astrocyte-neuronal networks during behaviors
Source: Neurophotonics. 2024 Nov 15;11(4):045010. doi: 10.1117/1.NPh.11.4.045010 (PMC11566604; doi:10.1117/1.NPh.11.4.045010)
Supplement: Supplementary file 1 [file NPh_011_045010_SD001.pdf]

Supplementary information

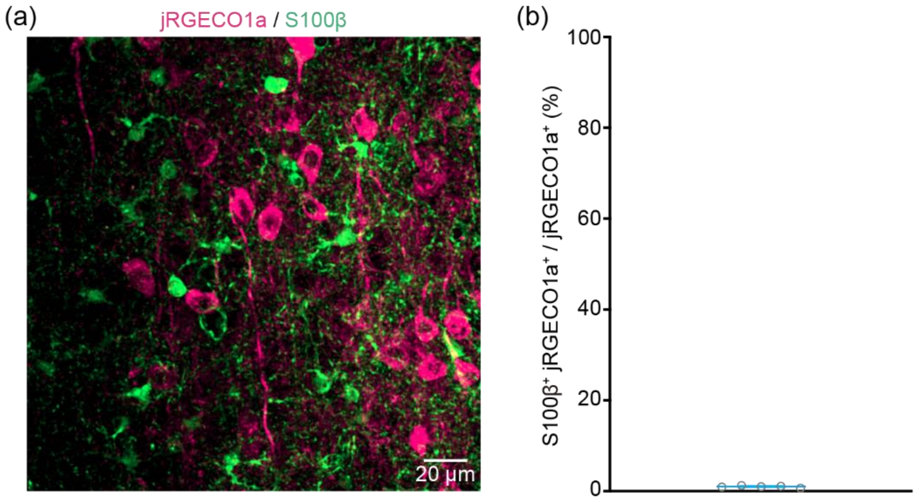

Supplementary Fig. 1 None of the jRGECO1a positive cells are labeled by S100β.

a The representative confocal image illustrates the immunostaining of jRGECO1a (magenta) and S100β (green) in the mouse cortex following virus injections. **b** The percentage of S100β and jRGECO1a co-labeled cells relative to all jRGECO1a positive cells (n = 5 mice).

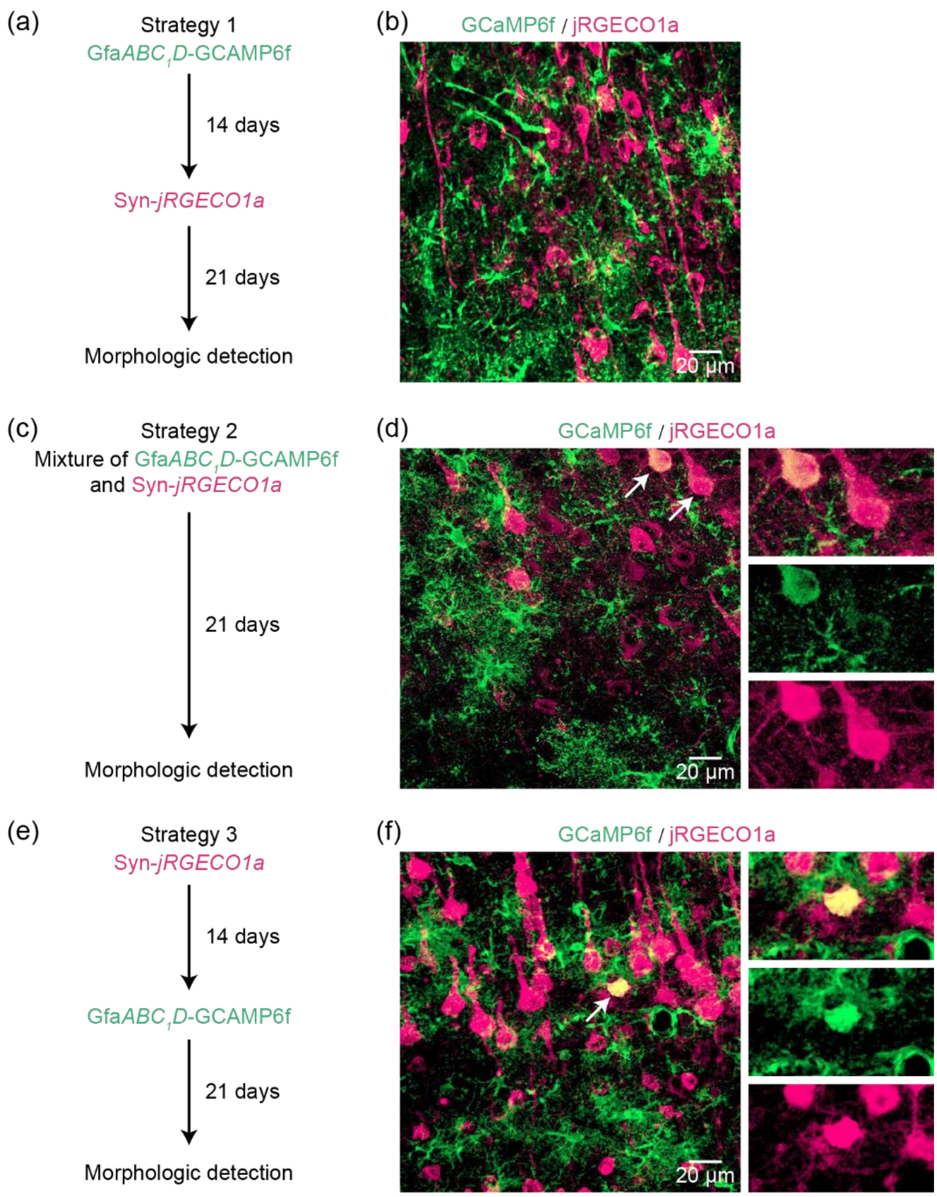

Supplementary Fig. 2 The expression of GCaMP6f and jRGECO1a was investigated using three distinct virus injection strategies. **a** Strategy 1: AAV5-GfaABC1D-cyto-GCaMP6f-SV40 was injected into the targeted brain region. Fourteen days later, we injected AAV9-hSyn-NES-jRGECO1a-SV40 into the same region. Twenty-one days following the AAV microinjections, the expressions of GCaMP6f and jRGECO1a were confirmed through post hoc histology. **b** The representative confocal image illustrates the expression of GCaMP6f (green) and jRGECO1a (magenta) in the mouse cortex following virus injections using strategy 1. **c** Strategy 2: AAV5-

GfaABC1D-cyto-GCaMP6f-SV40 and AAV9-hSyn-NES-jRGECO1a-SV40 were mixed in a 1:1 ratio and subsequently injected into the targeted brain region. Twenty-one days post-AAV microinjection, the expressions of GCaMP6f and jRGECO1a were confirmed through post hoc histological analysis. **d** Left, the representative confocal image illustrates the expression of GCaMP6f (green) and jRGECO1a (magenta) in the mouse cortex following virus injections using strategy 2. The cells co-labeled with GCaMP6f and jRGECO1a are indicated by white arrows. Right, the image of the cells, indicated by the white arrows, has been enlarged from the left image. **e** Strategy 3: AAV9-hSyn-NES-jRGECO1a-SV40 was injected into the targeted brain region. Fourteen days later, we injected AAV5-GfaABC1D-cyto-GCaMP6f-SV40 into the same region. Twenty-one days following the AAV microinjections, the expressions of GCaMP6f and jRGECO1a were confirmed through post hoc histology. **f** Left, the representative confocal image illustrates the expression of GCaMP6f (green) and jRGECO1a (magenta) in the mouse cortex following virus injections using strategy 3. The cell co-labeled with GCaMP6f and jRGECO1a is indicated by a white arrow. Right, the image of the cell, indicated by the white arrow, has been enlarged from the left image.

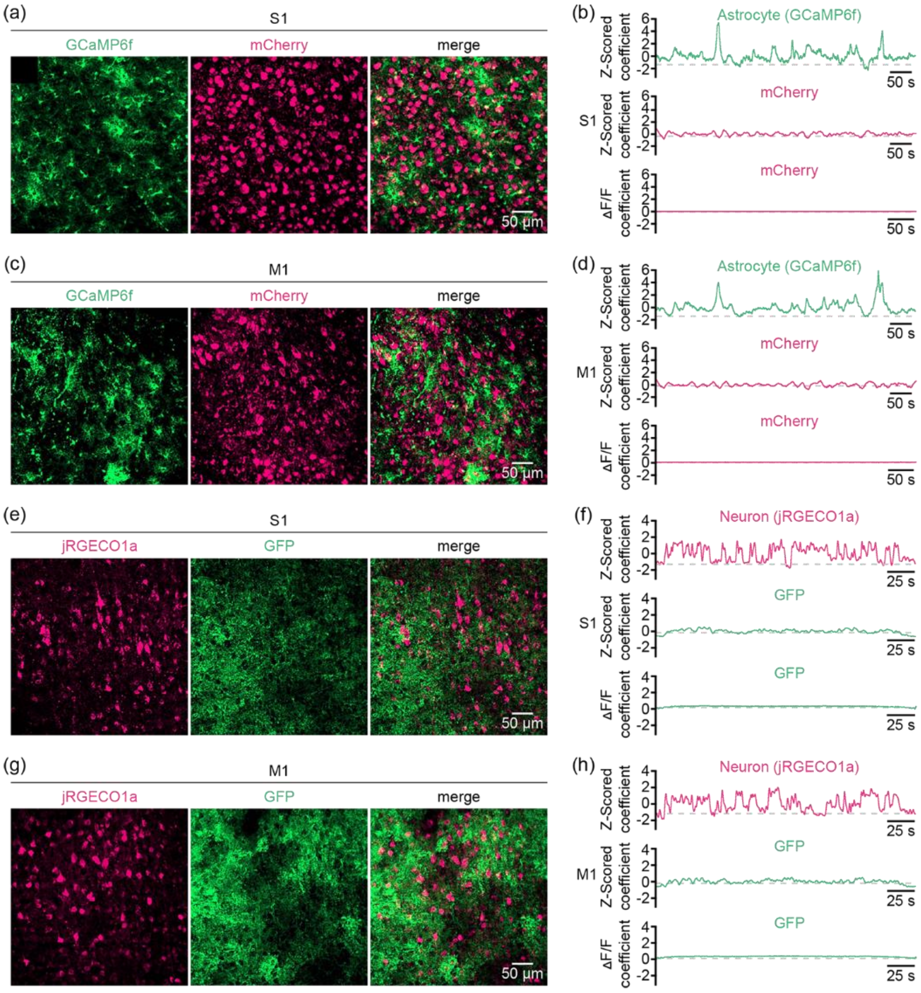

Supplementary Fig. 3 Simultaneous dual-color recordings were conducted using green GECI GCaMP6f expressed in astrocytes alongside mCherry in neurons, as well as red GECI jRGECO1a in neurons paired with GFP in astrocytes. a, c Expression of GCaMP6f in astrocytes and mCherry in neurons by virus injections of AAV5-GfaABC$_1$D-cyto-GCaMP6f-SV40 and AAV9-hSyn-NES-mCherry-WPRE-SV40 in S1 (a) and M1 (c) brain regions. **b, d** Normalized coefficients of GCaMP6f (top) and mCherry (middle), along with the ΔF/F of mCherry (bottom), are presented for the S1 (b) and M1 (d) brain regions during free behaviors. **e, g** Expression of jRGECO1a in neurons and GFP in astrocytes by virus injections of AAV9-hSyn-NES-jRGECO1a-WPRE-SV40 and AAV5-GfaABC$_1$D-PI-Lck-GFP-SV40 in S1 (e) and M1

(g) brain regions. **f, h** Normalized coefficients of jRGECO1a (top) and GFP (middle), along with the ΔF/F of GFP (bottom), are presented for the S1 (f) and M1 (h) brain regions during free behaviors.

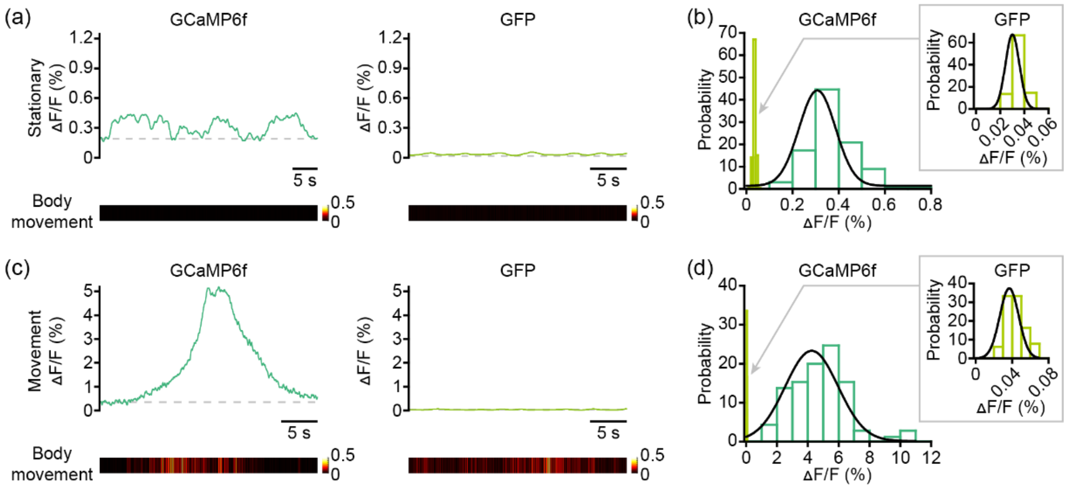

Supplementary Fig. 4 Astrocytic Ca^{2+} transients can be detected in both stationary and movement states. a, c Examples of recorded fluorescence traces are presented for two groups in stationary (a) or movement (c) states: the AAV5-GfaABC1D-cyto-GCaMP6f-SV40 group (left, turquoise) and the AAV5-GfaABC1D-PI-Lck-GFP-SV40-injected group (right, yellow-green). **b, d** The distributions of GCaMP6f fluorescence amplitudes (the turquoise histogram) and GFP fluorescence (the upper right yellow-green histogram, which is an expansion of the yellow-green line in the left histogram) are presented in both stationary (b) and movement (d) states. Both histograms fit Gaussian distributions. In stationary state, the mean values were 0.38% ΔF/F in the GCaMP6f group and 0.035% ΔF/F in the GFP group. In movement state, the mean values observed were 4.82% ΔF/F in the GCaMP6f group and 0.043% ΔF/F in the GFP group.

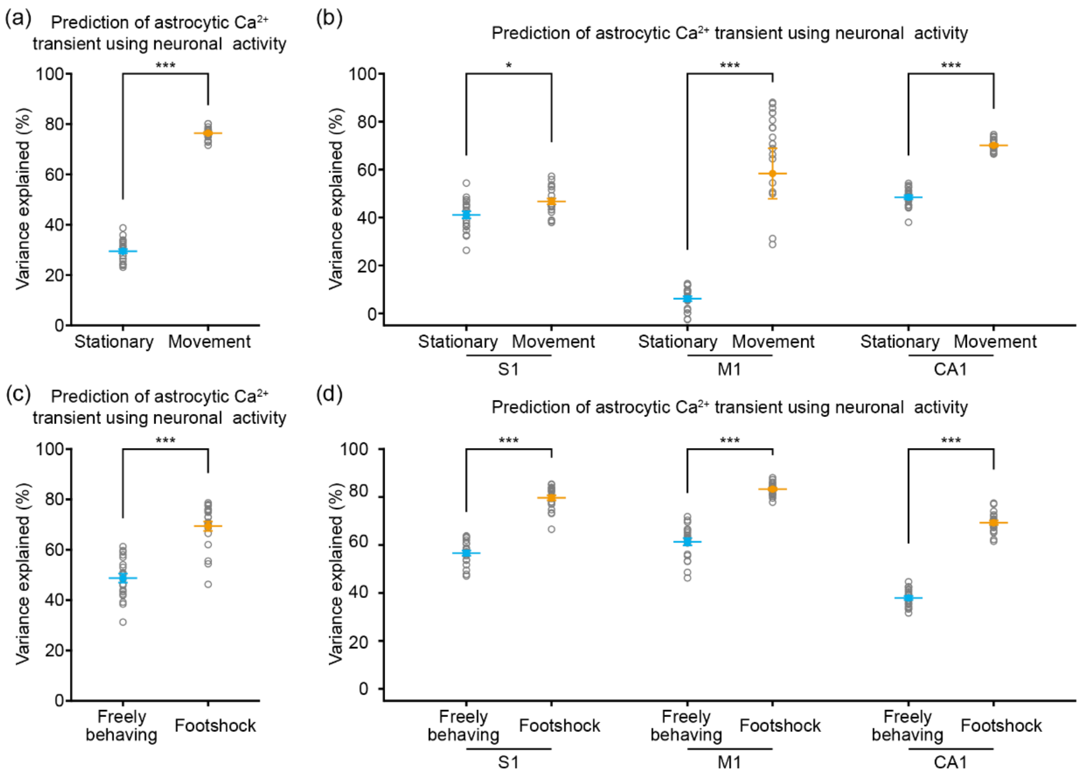

Supplementary Fig 5 Analysis of the contributions of neuronal activity to astrocytic Ca^{2+} transients across various brain regions. a A Random Forest Regression model was trained (95.83% of data, stationary: 4487 samples, movement: 4813 samples) to predict (4.17% of data, stationary: 195 samples, movement: 209 samples) astrocytic Ca^{2+} transients accurately in cortex during stationary (mean r^2= 0.30 ± 4.12E-02 std, n = 24 cross-validations) and movement states (mean r^2 = 0.77 ± 2.44E-02 std, n = 24 cross-validations), P = 2.88E-09, Z = -5.9385, ***P < 0.001; Wilcoxon' s rank-sum test. **b** A Random Forest Regression model was trained (S1: 95.0% of data, stationary: 1513 samples, movement: 2180 samples, M1: stationary: 95.0% of data, 3310 samples, movement: 94.74% of data, 2100 samples, CA1: 95.0% of data, stationary :1858 samples, movement: 3695 samples) to predict (S1: 5.0% of data, stationary: 79 samples, movement: 114 samples, M1: stationary: 5.0% of data, 174 samples, movement: 5.26% of data, 117 samples, CA1: 5.0% of data, stationary: 97

samples, movement: 194 samples) astrocytic Ca^{2+} transients accurately in S1, M1 and CA1 during stationary (S1: mean r^2 = 0.41 ± 6.74E-02 std, M1: mean r^2= 0.06 ± 4.72E-02 std, CA1: mean r^2 = 0.48 ± 3.87E-02 std, n = 20 cross-validations) and movement (S1: mean r^2 = 0.47 ± 5.69E-02 std, n = 20 cross-validations, M1: mean r^2= 0.68 ± 1.79E-01 std, n = 19 cross-validations, CA1: mean r^2 = 0.70 ± 2.53E-02 std, n = 20 cross-validations) state (stationary vs movement, S1: 1.86E-02, Z = -2.3534, M1: 9.37E-08, Z = -5.3385, CA1: P = 6.30E-08, Z = -5.4100, ***P < 0.001, *P < 0.05; Wilcoxon' s rank-sum test) . **c** A Random Forest Regression model was trained (95.0% of data, freely behaving: 589 samples, footshock: 589 samples) to predict (5.0% of data, freely behaving: 31 samples, footshock: 31 samples) astrocytic Ca^{2+} transients accurately in cortex during freely behaving (mean r^2= 0.49 ± 8.29E-02 std, n = 20 cross-validations) and footshock (mean r^2= 0.69 ± 8.52E-02 std, n = 20 cross-validations) states, P = 1.00E-06, Z = -4.8420, ***P < 0.001; Wilcoxon' s rank-sum test. **d** A Random Forest Regression model was trained (S1: 95.0% of data, freely bahaving: 10236 samples, footshock: 2508 samples, M1: 95.0% of data, freely bahaving: 7829 samples, footshock: 2508 samples, CA1: 95.0% of data, freely behaving: 9174 samples, footshock: 2508 samples) to predict (S1: 5.0% of data, freely behaving: 538 samples, footshock: 132 samples, M1: 5.0% of data, freely behaving: 412 samples, footshock: 132 samples, CA1: 5.0% of data, freely behaving: 482 samples, footshock: 132 samples) astrocytic Ca^{2+} transients accurately in S1, M1 and CA1 during freely behaving (S1: mean r^2= 0.57 ± 4.98E-02 std, M1: mean r^2= 0.61 ± 7.06E-02 std, CA1: mean r^2= 0.38 ± 3.79E-02 std, n = 20 cross-validations) and footshock (S1: mean

r^2= 0.80 ± 4.93E-02 std, M1: mean r^2= 0.83 ± 2.70E-02 std, CA1: mean r^2= 0.69 ± 4.19E-02 std, n = 20 cross-validations) states (stationary vs movement, S1: P = 6.30E-08, Z = -5.4100 , M1: P =6.30E-08 , Z = -5.4100 , CA1: P = 6.30E-08, Z = -5.4100, ***P < 0.001; Wilcoxon' s rank-sum test) .
